# QuickProbs—A Fast Multiple Sequence Alignment Algorithm Designed for Graphics Processors

**DOI:** 10.1371/journal.pone.0088901

**Published:** 2014-02-25

**Authors:** Adam Gudyś, Sebastian Deorowicz

**Affiliations:** Institute of Informatics, Faculty of Automatic Control, Electronics and Computer Science, Silesian University of Technology, Gliwice, Poland; The University of North Carolina at Charlotte, United States of America

## Abstract

Multiple sequence alignment is a crucial task in a number of biological analyses like secondary structure prediction, domain searching, phylogeny, etc. MSAProbs is currently the most accurate alignment algorithm, but its effectiveness is obtained at the expense of computational time. In the paper we present QuickProbs, the variant of MSAProbs customised for graphics processors. We selected the two most time consuming stages of MSAProbs to be redesigned for GPU execution: the posterior matrices calculation and the consistency transformation. Experiments on three popular benchmarks (BAliBASE, PREFAB, OXBench-X) on quad-core PC equipped with high-end graphics card show QuickProbs to be 5.7 to 9.7 times faster than original CPU-parallel MSAProbs. Additional tests performed on several protein families from Pfam database give overall speed-up of 6.7. Compared to other algorithms like MAFFT, MUSCLE, or ClustalW, QuickProbs proved to be much more accurate at similar speed. Additionally we introduce a tuned variant of QuickProbs which is significantly more accurate on sets of distantly related sequences than MSAProbs without exceeding its computation time. The GPU part of QuickProbs was implemented in OpenCL, thus the package is suitable for graphics processors produced by all major vendors.

## Introduction

Multiple sequence alignment (MSA) is an essential task in molecular biology. It is performed for both, nucleotide and protein sequences. Its field of applications covers phylogenetic analyses, gene finding, identification of functional domains, prediction of secondary structures, and many others. Rapidly increasing size of sequence databases allowed by the development of high throughput sequencing technologies provides biologists with the opportunity to analyse in silico enormous sets of data. Hence, the constant pressure for developing more accurate and faster MSA algorithms. As multiple sequence alignment problem is NP-hard [1], [2], exact methods are infeasible for practical applications due to excessive computation time. Therefore, many heuristics have been developed including progressive [3], iterative [4], or hidden Markov model-based [5] strategies. One of the most popular multiple sequence alignment software is ClustalW [6]. It is a classic representative of progressive algorithms, and works according to the scheme:

1. Estimate evolutionary distances between all pairs of sequences.
2. Build a guide tree on the basis of the distances.
3. Align sequences in the order described by the tree.

Calculation of an evolutionary distance is done either by performing pairwise sequence alignment between sequences (default mode) or by employing 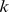-tuple matching (fast mode).

Many researches aimed at refining ClustalW accuracy by extending the idea of progressive alignment. An important breakthrough was the introduction of T-Coffee algorithm [7] which incorporated a consistency-based objective function. The principle was employing knowledge of some symbols being aligned from all pairwise alignments and was confirmed to improve significantly quality of a final result. Other techniques acquired by MSA algorithms include identification of homologous regions using fast Fourier transform (MAFFT [8]) or iterative refinement of a final alignment (MUSCLE [9]). There is a group of methods that improved calculation of a pairwise alignment by using suboptimal alignments. These are Probcons [10] and Probalign [11] which compute posterior probability matrices for all pairs of sequences using pair hidden Markov models (pair-HMMs) and partition functions, respectively. Both of them are also equipped in a consistency scheme which makes them very accurate. Recently published MSAProbs algorithm combines pair-HMM and posterior function approaches with a consistency transformation and an iterative refinement leading to the highest quality amongst all presented packages [12]. However, experiments show that even most accurate methods fail to find a proper alignment when analysed sequences are distantly related, particularly in so called ‘twilight zone’, when sequence similarity drops below 30%. Some algorithms addressed this issue by introducing to the alignment procedure additional knowledge. E.g., 3D-Coffee extends T-Coffee by using mixture of pairwise sequence and structure alignments [13]. MSACompro introduces to MSAProbs pipeline secondary structures, residue-residue contact maps, and solvent accessibility which elevates accuracy [14].

Our research, however, focuses on methods exploiting only sequences themselves. An important issue related to accurate MSA algorithms like T-Coffee, ProbCons, or MSAProbs is that superior results are produced at the cost of significant increase in complexity: all the above-mentioned methods are inferior to ClustalW in fast mode in terms of time and memory requirements. This is a serious disadvantage when processing large sequence sets, which is often the case, as it has been proven that introducing homologous sequences to MSA improves quality of a final result [15]. Moreover, there are applications which require huge number of multiple alignments to be computed. E.g., PhylomeDB database [16] gathers currently almost 1.9 million of MSAs. As alignment times varied from several seconds to several minutes, computation of all alignments required tens of months of CPU time [17]. Taking into account increasing availability of genomic and proteomic data, this number is expected to grow dramatically in close future. Hence, it is desirable to have algorithms able to align large sequence sets or perform large number of alignments in a reasonable time.

For aforementioned reason, some algorithms aimed at improving alignment quality without sacrificing time and memory efficiency of ClustalW. These are for example Kalign [8] and Kalign2 [19] which instead of 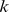-tuple matches employ respectively, Wu-Manber [20] and Muth-Manber [21] approximate string matching algorithms. Kalign2 turns out to be faster and more memory efficient than ClustalW in fast mode and also significantly more accurate (not as accurate as consistency-based methods, though). Kalign-LCS [22] further improved alignment accuracy and execution time by exploiting a bit-parallel longest common subsequence measure for distance calculation. The new version of MAFFT introduces PartTree algorithm [23] which allows a guide tree to be constructed without calculating all pairwise distances. A similar strategy was acquired by the recently published algorithm Clustal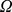 [24] that joins HMMs with mBed [25], a method of dimensionality reduction called sequence embedding. As a result, the number of pairwise alignments in both these packages is decreased from 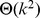 to 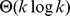 with respect to the number of sequences 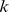. This makes MAFFT and Clustal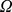 the only methods which are able to align tens of thousands of sequences in a reasonable time.

Nevertheless, experiments clearly show that if alignment quality is of paramount importance, consistency-based methods are out of competition. In order to overcome their greatest disadvantage, i.e., large execution times, many algorithms utilise multi-core architecture of modern CPUs. One of the best examples is MSAProbs which assessed on quad-core CPU turned out to be faster than its less accurate serial competitors like ProbCons or Probalign. Yet, parallelisation on central processors has its limitations. Nowadays, a typical desktop PC is equipped with four- or six-core CPU and to further decrease execution times, expensive multi-processor architectures have to be used. One of the ways of addressing this issue is using a potential of graphics processors in general purpose computing. Since computational power of current GPUs is more than order of magnitude greater than power of central processors, developing GPU-suited versions of algorithms has become popular in many computational demanding tasks also in bioinformatics. One must keep in mind, that differences in architectures of graphics processors and CPUs are fundamental. GPUs have thousands of cores, several types of memory and utilise massively parallel execution model. This makes designing algorithms customised for GPUs a challenging task that cannot be accomplished by adapting serial or parallel methods destined for CPU execution.

Heretofore, GPU customisation of multiple sequence alignment algorithms concerned mainly ClustalW [26], [27] and different variants of MSA problem like constrained MSA investigated by authors of this paper [28] or regular expression MSA [29]. The only attempt to parallelise on graphics processor an accurate, consistency-based multiple sequence alignment method was G-MSA [30], a variant of T-Coffee algorithm. The authors, however, focused on decreasing execution times and introduced some modifications that lowered quality of an output alignment. As a result, G-MSA turned out to be very fast (even 193 times faster than its predecessor), but inferior in terms of accuracy not only to original T-Coffee, but also to some non-consistency algorithms like MUSCLE. The aim of our research is different. We selected MSAProbs, the most accurate from existing MSA methods as our starting point and developed QuickProbs. It is a variant of MSAProbs algorithm suited for graphics processors preserving outstanding accuracy of its predecessor.

The algorithm executes on GPU the most time consuming parts of MSAProbs pipeline, i.e., the posterior probability matrices calculation and the consistency transformation. These stages are parallelised in MSAProbs on CPU with a use of OpenMP [31]. The parallelisation is, however, based on inter-task execution model which is unsuitable for graphics processors because of their massively parallel architecture. Therefore, GPU-specific algorithms for these stages had to be designed.

The posterior calculation stage executes dynamic programming methods like *forward-backward* algorithm for pair-HMMs or partition function calculation. The dynamic programming (DP) was a subject of GPU customisation multiple times, also in bioinformatics. It concerned pairwise sequence alignment [32]–[37], short read alignment [38], RNA folding [39], phylogeny [40], etc. Each application has some specific features that require suited algorithms, though. In MSAProbs these features include presence of several dependant DP layers, different storage patterns for different layers, a complex form of recursive expression making GPU code register-bound.

The consistency transformation stage incorporates a set of sparse matrix multiplications. There are algorithms and libraries for this task [41]–[43]. The multiplication procedure exploited by MSAProbs has however some specific features. Firstly, many multiplications of small matrices is performed, while previously published solutions are optimised for large matrices. Secondly, the consistency transformation does not allow new elements to be introduced to output matrices. Due to these reasons existing methods cannot be used directly for our aims.

QuickProbs uses new, graphics processor specific, intra-task parallel algorithms for both, posterior matrix calculation and consistency transformation. Additionally, we parallelised on CPU the alignment construction and refinement stage, which in MSAProbs is performed serially. As a result, our package is several times faster than MSAProbs. This allows user to process larger datasets in a reasonable time without sacrificing alignment quality. Additionally we present a tuned variant of method called QuickProbs-acc. It significantly outperforms MSAProbs in terms of accuracy on sets of distantly related sequences without exceeding its running times.

## Materials and Methods

### Problem formulation

Let 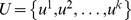 be the set of input sequences. Multiple sequence alignment problem consists in arranging sequences from 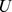 by putting gaps between symbols in the way that homologous residues are aligned together in columns. Homologous residues are those which share three dimensional structural position and diverge from common ancestral residue [44]. The problem of MSA is that for majority of cases it is impossible to identify a single correct alignment. This is because both structures and sequences evolve and some residues cannot be superposed in any way. This must be taken into account when assessing multiple sequence alignment algorithms. Therefore, a subset of key residues and core structural blocks that can be unambiguously aligned is identified and used for evaluation. The most commonly used assessment measures calculated on these regions are sum-of-pairs (SP) and total-column (TC) scores [45]. They denote percentage of properly aligned residue pairs and columns, respectively, and a reference alignment is required to compute them. In the case of real sequences it is usually constructed manually. If testing sets are generated synthetically with a use of evolution modelling software like ROSE [46], the reference alignment is built during artificial evolutionary process by the software itself.

### General purpose computing on GPU

Computational power of current graphics processors is several times greater than power of CPUs. Rapid development of programming interfaces like CUDA [47] or OpenCL [48] allows this power to be employed in general purpose computing. Due to this fact, designing algorithms customised for graphics processors has recently become an important method of speeding up analyses of large datasets as an alternative to using expensive multi-processor architectures based on CPUs. In QuickProbs, GPU computing is performed with a use of OpenCL library since, unlike CUDA, it is suitable for graphics processors produced by both major vendors, NVidia and AMD. Hence, in the following description we hold to the OpenCL nomenclature providing CUDA terms in parentheses.

The reason why designing algorithms suited for GPU execution is a challenging task is a great difference between architectures of central and graphics processors. Unlike CPUs that contain few cores, modern GPUs are composed of thousands of *processing elements* (*cores*) gathered in several *compute units* (*multiprocessors*). Processing elements within compute units operate according to a single instruction-multiple data or single program-multiple data paradigm. From logical point of view a GPU program (known as a *kernel* in both OpenCL and CUDA) consists of many *work-items* (*threads*) gathered in *workgroups* (*blocks*). An important fact is that synchronisation between work-items can be done only within a workgroup. Thus, matching the number and the size of workgroups for a particular task is a crucial issue when developing GPU-suited algorithms. After execution of a kernel, a hardware *scheduler* maps work-items in the way that a workgroup is executed on a single compute unit, while one unit can handle multiple workgroups. OpenCL does not specify how workgroups are run by hardware but in order to efficiently utilise computational power of GPU, knowledge of workgroup execution at the device level is necessary. The smallest amount of work that is physically performed on AMD GPUs consists of 64 work-items and is called a *wavefront*; on NVidia devices it has 32 items and is known as a *warp*. Therefore, it is important to make the group size multiplicity of these portions. There is no guarantee in which order wavefronts (warps) are executed—this is decided by the scheduler dynamically. An important consideration is that all work-items within that portion must share exactly the same execution path. A divergence in a wavefront (warp) caused, e.g., by the presence of conditional statements is realised by executing instructions from all paths with some work-items being masked when necessary. Hence, data dependant branching inside wavefronts (warps) increases kernel execution time and should be avoided. Another important issue when developing algorithms on GPU is providing sufficient *occupancy* of a device. In order to hide delays of arithmetic instructions and, most importantly, memory accesses it is recommended to invoke a few times more work-items than the number of processing elements. On modern devices this results in as much as 10^4^ work-items per kernel.

An important difference between CPU and GPU concerns memory architecture. GPU is equipped with few gigabytes of *global memory* which is an equivalent of main memory at CPU. Maximal throughput of global memory is several times greater than main memory bandwidth. However, it can be achieved only in the case of coalesced accesses when consecutive work-items utilise data from contiguous 128-byte area. In such situation whole portion of data can be read in one transaction. Since latency of global memory is large, non-coalesced accesses often result in lower bandwidth than at CPU. This issue has been partially solved in recent graphics processors by caching. Nevertheless, compared to CPUs which have several megabytes of cache per core, amount of cache on GPU is much smaller—hundreds of bytes per processing element. Another limitation concerning global addressing space is lack of virtual memory on GPU: programmer is responsible for fitting all necessary data in a limited storage area. Each compute unit contains additional amount (tens of kilobytes) of fast, directly addressable *local memory* (*shared memory*) which can be used for buffering frequently accessed data. Graphics processors contain much more registers than CPUs (tens of thousands per compute unit). The size of local memory and the number of registers allocated by work-items are important for execution time as they affect the maximum size of workgroup as well as GPU occupancy.

Concluding, graphics processor execution model differs significantly from CPU. Presence of thousands of cores at GPU device requires fine-grained algorithm parallelisation. Programmer needs to take care of coalesced accesses to global memory to maximise throughput. Careful use of local memory and registers is necessary to keep GPU occupancy at desired level. Finally, calculations should be organised in the way, that eliminates branching within the same wavefront (warp). As a result, algorithms destined for graphics processors often use different data structures and computation schemes than methods suited for central processors, even those exploiting multi-core architectures. Due to this fact algorithms for GPUs must be designed and implemented from the scratch rather than be adopted from CPU.

### MSAProbs algorithm principals

MSAProbs is currently the most accurate multiple sequence alignment software. It is a progressive strategy based on the following stages:

I. Calculation of posterior probability matrices and distances for all pairs of sequences.

II. Construction of a guide tree upon distances and calculation of sequence weights.

III. Performing weighted consistency transformation on all posterior matrices.

IV. Building final alignment using the guide tree and posterior matrices followed by the iterative refinement.

**Figure 1:**
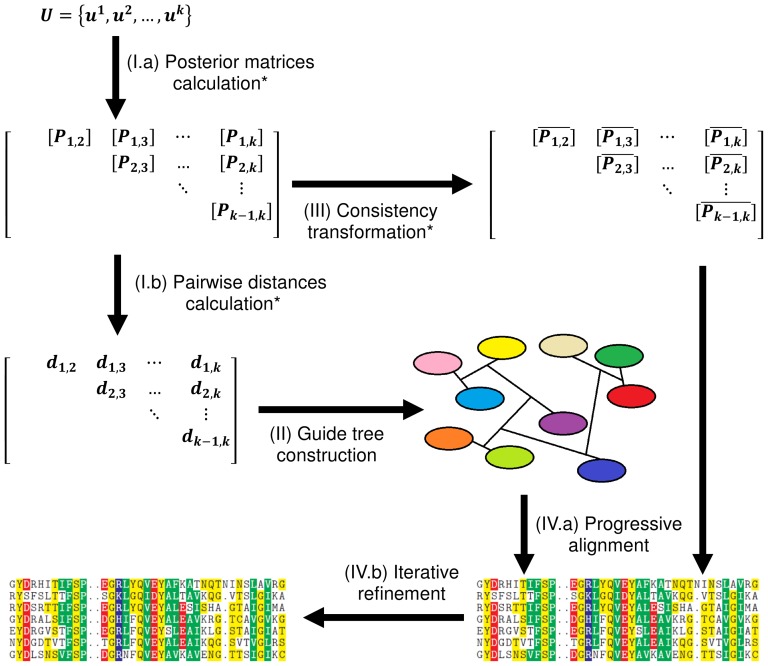
MSAProbs algorithm computation scheme. Stages denoted with (*) are run in parallel with a use of OpenMP.

The algorithm scheme can be found on Figure 1. Stages I and IV were divided in the diagram into two sub-stages in order to present data dependencies. At the beginning, the algorithm computes posterior probability matrices (stage I.a) which contain detailed information of residue alignments for all sequence pairs and are used for distances calculation (stage I.b). Many progressive algorithms estimate distance between two sequences on the basis of a maximum probability alignment computed with Viterbi algorithm [49]. The main disadvantage of this approach is that it takes into account only one possible alignment of the sequences, thus it is error-prone. In contrast, MSAProbs computes for each pair of sequences a posterior probability matrix which is further used for calculation of a maximum expected accuracy alignment [44]. As it takes advantage of suboptimal alignments, it improves quality of results. As in all progressive methods, distances are required for guide tree construction (stage II). This is done with a variant of UPGMA algorithm [50]. Before MSAProbs constructs a final alignment it performs so called consistency transformation (stage III). Single posterior matrix computed in stage I contains information only from pairwise alignments of two sequences, which may cause errors in the result if some residues are aligned improperly. The consistency transformation relaxes posterior matrices over other sequences. Thanks to this, matrices contain information of residue alignment from pairwise alignments of all sequences. This reduces the probability of misaligning some symbols during construction of the final result. Number of consistency iterations is one of the algorithm parameters. Afterwards, sequences are aligned greedily in the order described by the tree (stage IV.a). At each tree node, a profile-profile alignment is performed with a use of relaxed posterior matrices calculated previously. When the alignment is constructed, the refinement stage begins (stage IV.b). It randomly splits the alignment horizontally and realigns resulting profiles. Thanks to this, the algorithm is able to remove some errors introduced in previous stages. The number of refinement iterations is also a parameter. Summing up, MSAProbs has three features crucial for its accuracy:

- exploiting maximum accuracy criterion during pairwise alignments,
- relaxing posterior matrices over other sequences,
- performing the iterative refinement.

First two methods decrease probability of misaligning given pair of residues by utilising information from suboptimal pairwise alignments and alignments of other sequences. If misalignment occurs, third feature gives the opportunity to correct it.

Predominance of MSAProbs over previously published methods is obtained at the cost of computation time. The most time consuming stages are those performed for all pairs of sequences, that is the posterior matrix calculation (I) and the consistency transformation (III). The worst-case time complexities of these stages are 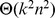 and 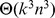, respectively, for sequences of length 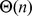. In order to decrease computation time, those operations were chosen in MSAProbs to be parallelised using OpenMP. Following subsections describe all MSAProbs stages with a special stress put on stages I, III, and IV which were redesigned in QuickProbs.

**Posterior matrix calculation.** This stage of MSAProbs consists of calculating set of posterior probability matrices for all pairs of sequences 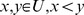, where ‘<’ indicates an ordering relation of sequences within 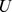. Let 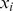 and 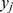 denote 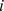
'th and 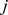
'th symbols of 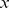 and 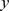 sequences, respectively. Elements of posterior probability matrix 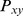 express 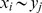, that is a probability of symbols 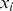 and 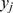 being aligned in the true alignment of 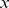 and 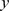. In MSAProbs, 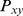 elements are root mean squares (RMS) of posterior probabilities calculated using pair hidden Markov models (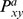) and partition function (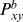): 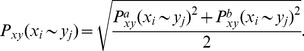
(1)


After posterior matrix computation, the Needleman-Wunsch algorithm [51] with no gap penalties is applied on 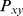 in order to determine distance 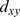 between sequences. As majority of 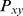 elements are close to 0, in order to save space and accelerate the consistency transformation, posterior matrices are translated to a sparse form by filtering out all elements less than 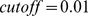. For convenience, sparse representation of 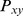 matrix will be referred to as 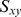. Let us denote the calculation of particular 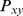 and 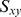 as a posterior task, resulting in 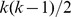 tasks to be processed. Parallelism of posterior stage in MSAProbs is provided by distributing tasks among several OpenMP threads. As each thread calculates one or more tasks, this parallelisation model can be referred to as *inter-task*.

**Tree construction and sequence weighting.** The guide tree construction in MSAProbs is performed with a use of UPGMA algorithm. After that sequences are weighted in the order described by the tree using ClustalW weighting scheme. The weight of sequence 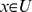 will be denoted as 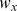.

**Consistency transformation.** The consistency transformation stage in MSAProbs relies in relaxing all posterior matrices calculated in stage I over sequences from 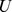. The transformation is repeated 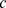 times (2 by default). The procedure of updating 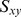 matrix will be referred to as a relaxation task and requires a set of 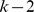 sparse-sparse matrix multiplications. It is done according to the formula: 
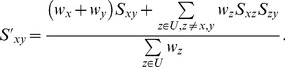
(2)


An important note is that MSAProbs does not allow any new elements to be introduced to 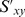 matrix. More precisely, it may happen, that in a row of 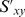 an element appears such that there is no element with the same column number in a corresponding row of 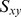. Such elements are filtered out from 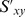 after multiplication. Moreover, the elements with values below 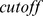 are dropped. As a result 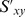 always has less or equal number of elements than 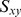. MSAProbs performs consistency for 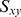 matrices such that 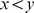, i.e., the input and output of the relaxation procedure is only an upper triangle of a matrix table. As a consequence, the algorithm chooses one of the three versions of multiplication procedure depending on the ordering of 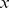, 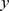, and 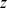 sequences, i.e., 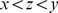, 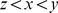, and 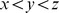. Matrices that are being relaxed are temporarily stored in the dense form 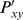 and translated back to the sparse representation 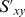 at the end of the task. After all of the tasks finish, 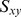 matrices are replaced by 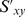. Parallelism in the consistency transformation is provided, similarly to stage I, by inter-task execution: an OpenMP thread is responsible for relaxation of one or more 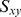 matrices. The posterior matrices after performing 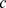 consistency transformations will be referred to as 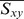 (on Figure 1 we neglect the fact that matrices are stored in the sparse form, thus 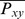 symbol is used).

**Final alignment construction and refinement.** The last stage of all progressive methods is the computation of final alignment according to the guide tree constructed during stage II. At each tree node a weighted profile-profile alignment (referred to as a progressive step) is performed. When aligning two profiles 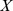 and 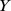 posterior probability matrix 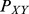 is calculated using 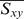 matrices for all pairs of sequences 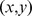 such that 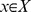 and 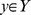. Then, the profile-profile alignment is constructed with a use of posterior probabilities 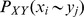 of aligning 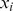 and 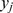 profile residues. This is done according to the following dynamic programming recursion: 
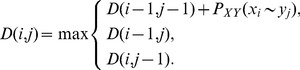
(3)


Calculation of the final alignment is followed by the refinement, one of the classic techniques of progressive methods. In MSAProbs it is called a post-processing stage. It was designed as a solution for one of the greatest problems of progressive strategies—wrong choice of sequences or profiles to be aligned in early steps of the final result construction. Such misalignments cannot be corrected in the following progressive steps and affect overall accuracy of the method. The refinement tries to solve this problem by splitting final alignment horizontally into two random profiles. Then, the columns containing only gaps are removed and the profiles are realigned in the same way as in the final alignment stage. The aforementioned procedure is repeated 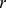 times (10 by default). The more refinement iterations, the greatest the chance of removing errors introduced during progressive construction.

In MSAProbs the final alignment and refinement procedures are executed serially.

### QuickProbs algorithm

The main goal of our study was to speed up the most time-consuming stages of MSAProbs (posterior matrices calculation and consistency transformation) by redesigning them for GPU execution using OpenCL. Additionally, we decided to parallelise on CPU the last stage of MSAProbs algorithm which previously was implemented in a serial manner. This is because the construction and refinement of final alignment, if executed serially, would be the most time consuming part of QuickProbs. In the following subsections we present how stages I, III, and IV were modified in QuickProbs. The separate subsection is devoted to QuickProbs-acc, a specialised variant of our package which aims at improving MSAProbs quality.

**Posterior matrix calculation.** The posterior matrix calculation in QuickProbs is, similarly to MSAProbs, based on tasks. However, massively parallel architecture of graphics processors requires smaller portions of work to be done by a work-item. Therefore, it was assumed that each task is processed by a single workgroup. That computation scheme can be denoted as *intra-task* parallelisation (one task—many work-items). As work-items within workgroup can synchronise and exchange data through local memory, they are able to properly handle data dependencies present in a task. Another important difference is that modifications made in QuickProbs algorithm at the consistency stage result in the necessity of calculating 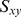 for all 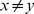, not only 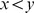. As 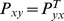, it is sufficient to calculate in the dense form only one of these two matrices (either 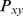 or 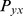) and then transform it to both 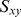 and 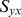. Therefore, QuickProbs posterior task takes as an input two sequences 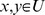 and consists of three steps:

1. calculate dense posterior matrix 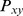 (or 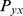, depending on sequence lengths) and pairwise distance 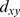,
2. build sparse matrix 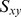 on the basis of 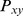 (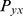),
3. build sparse transposed matrix 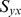 on the basis of 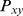 (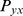).

Let 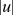 indicate the length of sequence 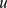 increased by 1. Size of 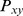 posterior matrix is 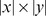 (sequences 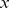 and 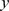 correspond to vertical and horizontal dimensions, respectively). QuickProbs parallelisation scheme assumes that each work-item in a workgroup calculates a single column of posterior matrix. Thus, the width 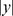 of 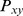 matrix that can be processed on GPU is limited by the maximum number of work-items in a workgroup 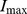 for a particular device. In current graphics processors like Radeon 7970 or GeForce 680 this limit is 1024 which is sufficient for majority of multiple sequence alignment problems. If 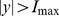 and 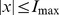, the algorithm calculates 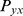 matrix instead of 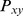. However, if 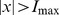 and 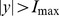, the task cannot be calculated at GPU and is scheduled for CPU execution.

At the beginning of posterior calculation stage, descriptions of all posterior tasks are prepared. As each task consists in computing two sparse matrices 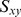 and 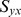, there are 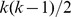 tasks in total. The orientation of a dense posterior matrix to be calculated by a task depends on lengths of sequences. Namely, the shorter sequence corresponds to the horizontal dimension (task width) and the longer to the vertical dimension (task height). Tasks of width exceeding 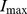 are scheduled for CPU execution. The rest is processed at GPU in batches. An important feature is that workgroups from different batches are scheduled independently. Therefore, workgroup resources, i.e., the number of work-items and the amount of local memory to be reserved are determined by the task with the largest horizontal dimension. Thus, the smaller divergence of task widths in a batch, the less GPU resources are wasted. Generally, the number of tasks in a batch is limited by the size of global memory available for a particular device 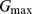 (detailed description of task memory requirements is given later). Nevertheless, in order to produce smaller and less divergent batches, we lowered global memory limit to 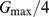 which produced best results in our preliminary experiments. Tasks are sorted in a descending order according to the width. Then, consecutive tasks are added to the current batch as long as its global memory requirement does not exceed 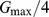. If that happens, a new batch is created and procedure repeats until no task remains. Finally, to reduce width divergence in batches and improve GPU utilisation, QuickProbs transposes all tasks in a batch with heights smaller than the number of work-items in the workgroup (it does not affect required number of work-items but decreases columns length).

**Figure 2:**
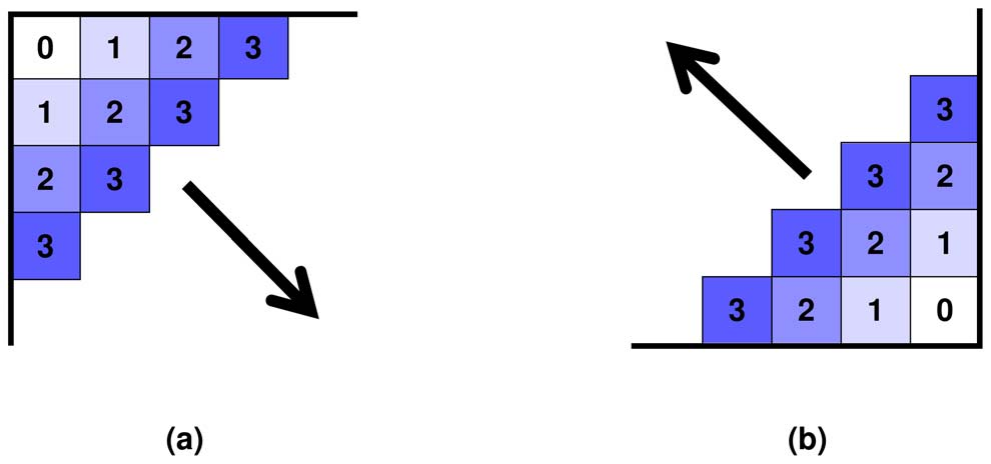
Scheme of traversing dynamic programming matrices in forward (a) and reverse (b) passes. Cells contain numbers of iterations in which they are processed.

Step (1) of the posterior task consists in computation of matrix 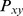 as a root mean square of 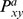 and 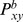 matrices. Pair-HMM calculates posterior probability matrix 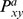 using *forward-backward* algorithm [44]. The procedure consists of two dynamic programming passes. They calculate matrices of forward and backward probabilities which are afterwards combined to form 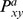. 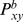 is calculated using values of partition functions of forward and reverse 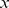 and 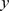 alignments as in [11]. The procedure, analogously to pair-HMM, requires two dynamic programming passes. At the end of step (1) 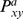 and 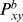 are joined to form 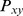 and an additional DP pass calculates pairwise distance 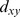 from 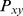. In general, dependencies in the DP matrices in the above-mentioned procedures are 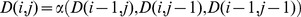
(4)


and 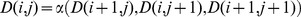
(5)


for forward and reverse passes, respectively, with different 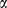 functions. The more detailed information about forms of a dynamic programming recursions used in 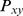 and 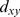 calculations is presented in Table 1. According to QuickProbs parallelisation scheme, each work-item within a workgroup calculates a single column of posterior matrix. Therefore, dynamic programming procedure must be executed according to an anti-diagonal pattern as presented in Figure 2. The whole matrix is processed in 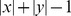 iterations in the way that 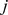
'th thread idles for 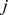 and 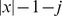 first iterations in forward and reverse passes, respectively. The idea of the dynamic programming computation scheme exploited by QuickProbs is presented in Table 2.

**Table 1:** Algorithm 1.

**Input:**	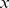 , 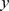 —sequences for which posterior matrix is to be calculated, 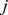 —work-item identifier (column number).
**Output:**	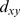 —distance between 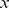 and 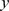 sequences, 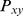 —output posterior matrix in the dense form.
1:	Initialise auxiliary matrices 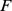 and 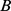 of size 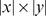 .
2:	Posterior function forward pass: 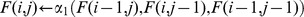
3:	Posterior function reverse pass: 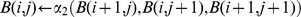
4:	Combine forward and reverse matrices: 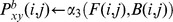
5:	Pair-HMM forward pass: 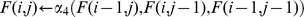
6:	Pair-HMM backward pass: 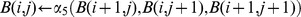
7:	Combine forward and backward matrices: 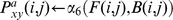
8:	Calculate final posterior matrix: 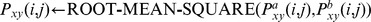
9:	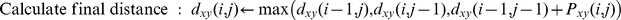
10:	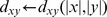

Pseudo-code of the posterior matrix calculation procedure. Statements show only general form of data dependencies. Procedure requires execution of 6 anti-diagonal passes (lines 2, 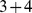, 5, 6, 7, and 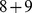). 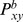 is computed first due to greater memory requirements.

**Table 2:** Algorithm 2.

**Input:**	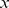 , 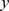 —input sequences, 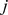 —work-item identifier (column number).
**Output:**	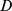 —matrix to be calculated.
1:	**function** forward
2:	Initialise 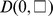 and 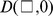
3:	**for** 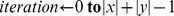 **do**
4:	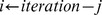
5:	**if** 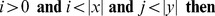
6:	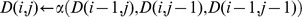
7:	**end if**
8:	synchronise
9:	**end for**
10:	**return** 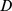
11:	**end function**
12:	**function** reverse
13:	Initialise 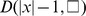 and 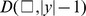
14:	**for ** 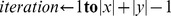 **do**
15:	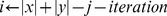
16:	**if** 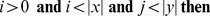
17:	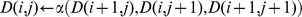
18:	**end if**
19:	synchronise
20:	**end for**
21:	**return** 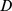
22:	**end function**

Pseudo-code of the generalised dynamic programming forward and reversed passes. 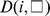 and 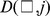 indicate 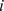
'th row and 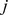
'th column of 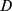 matrix.

**Figure 3:**
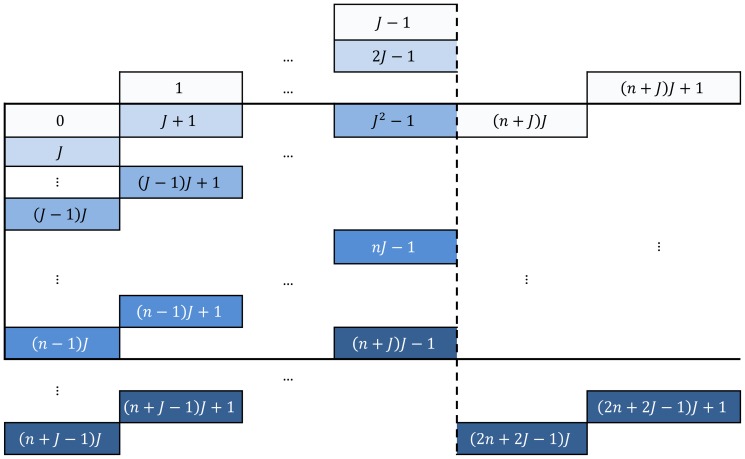
Jagged pattern of storing dense matrices in graphics device global memory. The size of jag is denoted as 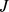.

Note, that all intermediate matrices needed for 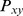 calculation must be stored in device global memory. Due to fact that dynamic programming requires accessing whole matrices with very limited data reuse, it will not take advantage of caching. Therefore, providing best possible global memory access pattern is a key issue for algorithm execution time. Taking into account anti-diagonal calculation scheme, simple row-major matrix representation results in a non-coalesced access which drastically decreases algorithm performance (each matrix cell is accessed in a separate transaction). Hence, a jagged memory layout of matrices elements was proposed (see Figure 3) in which consecutive work-items access consecutive memory cells resulting in perfect coalescing. Let 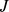 indicate the size of the jag. The number of elements necessary for storing matrix for sequences 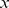 and 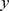 in the jagged form is 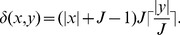
(6)


For convenience an *element* is used as a basic memory unit in the paper. Sometimes it is referred to as a *dense element* to distinguish it from *sparse elements* defined later. Pair-HMM used in MSAProbs contains 5 states. MSAProbs stores whole DP matrices for all states resulting in 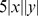 elements for both, forward and backward procedures. However, as we are only interested in full matrix at 0'th state, it is sufficient to store only two consecutive rows of layers 1–4 (they are required for the dynamic programming recursion). This reduces memory requirements for 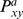 calculation to 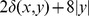, which is an important improvement, as it allows rows from layers 1–4 to be stored in fast local memory. Combination of forward and backward matrices can be done in place. The similar situation occurs in the case of partition function calculation. The difference here is that intermediate results are stored in a double floating-point precision. Therefore, in order to save space, reverse and combination passes are merged. The total memory footprint of calculating 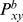 is 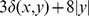 elements. The calculation of a root mean square of 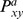 and 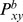 is done in place so it introduces no additional memory overhead. Eventually, the computation of 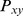 requires 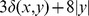 elements in total. Note that 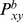 has to be computed first and stored so 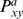 can be calculated in the remaining space.

Steps (2) and (3) of the posterior task transform dense 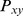 matrix to sparse representations 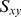 and 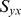, respectively. The main component of 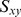 is an array of so-called *sparse elements* denoted as 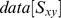. Each sparse element is a pair containing a column number and a floating-point value of posterior probability, referred to as 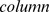 and 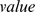, respectively. Additionally, there are two vectors storing sizes of rows and starting indices of rows in the sparse elements array. They are denoted as  and 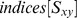. The 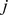
'th sparse element in 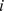
'th row of 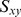 matrix is denoted as 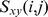 and can be accessed by using the formula 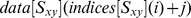. Such representation has its impact on a sparse generation procedure. Namely, in order to store 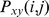 element in array 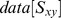 the number of non-zeros are in all previous rows and part of 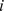
'th row before 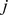
'th element have to be known. The similar situation is during 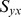 generation but instead of rows we are interested in 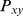 columns. Therefore, the transformation to the sparse form is done in two passes. In the first pass the number of non-zero elements in 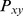 rows (or columns) is determined and stored in 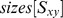 (or 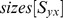). In the second pass 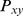 rows (columns) are transformed to the sparse form concurrently producing final 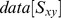 (or 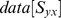) arrays. As 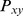 matrix is stored in the jagged form, 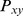 is traversed anti-diagonally during both passes of 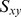 (or 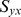) generation. The procedure of transforming 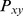 dense matrix to sparse 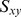 form is presented in Table 3.

**Table 3:** Algorithm 3.

**Input:**	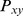 —dense matrix to be translated to sparse form, 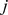 —work-item identifier (column number)
**Output:**	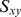 —output sparse matrix described by 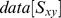 , 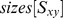 and 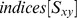 arrays.
1:	Initialise in parallel 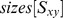 vector with 0.
2:	synchronise
3:	**for** 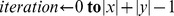 **do** 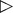 First pass: fill sizes vector.
4:	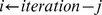
5:	**if** 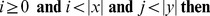
6:	**if** 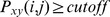 **then**
7:	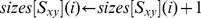
8:	**end if**
9:	**end if**
10:	synchronise
11:	**end for**
12:	**if** 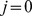 **then**
13:	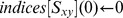
14:	**for** 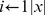 **do**
15:	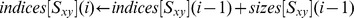
16:	**end for**
17:	**end if**
18:	synchronise
19:	Copy in parallel 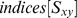 to auxiliary vector 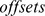 .
20:	synchronise
21:	**for** 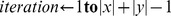 **do** 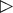 Second pass: fill sparse elements array.
22:	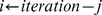
23:	**if** 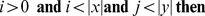
24:	**if** 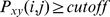 **then**
25:	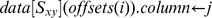
26:	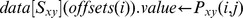
27:	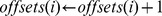
28:	**end if**
29:	**end if**
30:	synchronise
31:	**end for**

Pseudo-code of the sparse matrix generation procedure.

If column numbers and indices are of the same size as matrix elements, storing 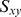 requires at most 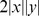 elements for 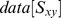 (if none of 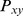 elements is below 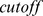) and 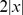 elements for 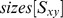 and 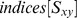. Calculations of 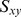 and 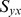 matrices are serialised, i.e., the algorithm executes step (2), reads the resulting sparse matrix from device memory, executes step (3), and reads the result once again. Therefore, the same memory space can be reused for both 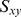 and 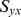 matrices. Taking into account space needed for storing the input dense matrix, total memory requirements for executing steps (2) and (3) is 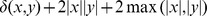.

Assuming that 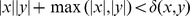, execution of a single task requires in total 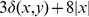
(7)


elements in device memory. The space for storing posterior layers and output sparse matrices, i.e., 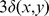 elements, is allocated in device global memory. Auxiliary rows consisting of 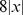 elements are placed in local memory to reduce transfer overheads. Let 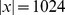 which is the maximum size of a workgroup that can be processed by modern GPUs. For such tasks the local memory requirement is 8,192 elements which equals 32 KB assuming 32-bit floating-point values. The last few generations of graphics processors are equipped in such amount of local memory. Thus storing auxiliary rows in local memory does not limit the size of datasets that can be processed by QuickProbs.

**Consistency transformation.** QuickProbs version of consistency transformation differs from its predecessor. First of all, as GPU computations are strongly memory limited, QuickProbs operates directly on sparse representations. As a result, the elements of 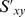 that do not appear in 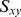 are discarded during multiplication procedure, not after, as in MSAProbs. Secondly, in order to eliminate divergence in kernel executions only one version of the sparse matrix multiplication procedure is implemented. This, however, requires all 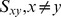 matrices at the input of the consistency transformation. These are provided by the modified stage I of QuickProbs. Additionally, as the output of 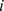
'th consistency iteration is also the input to 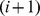
'th, all 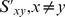 must be computed in the consistency stage. Since computational effort of matrix transposition is irrelevant with respect to the relaxation, QuickProbs calculates on GPU 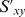 matrices only for 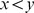 and then transposes them in parallel on CPU with a use of OpenMP generating 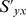. The last difference concerns parallelisation scheme, which was changed to intra-task, i.e., each task is analysed by entire workgroup. As previously, after each transformation 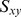 matrices are replaced by 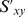. Posterior matrices after performing all 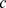 consistency transformations will be referred to as 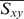.

At the beginning of the consistency transformation QuickProbs generates descriptions of all 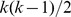 relaxation tasks. In order to reduce GPU global memory requirements the relaxation is performed in batches, similarly to the posterior matrices calculation. Namely, set of sequences 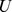 is divided in equally-sized subsets 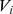. As a result, table of 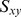 matrices can be divided in square sectors denoted as 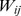. Afterwards, the execution of two nested loops begins. The outer loop iterates over sectors 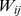 to be computed. As QuickProbs calculates on GPU 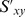 matrices only for 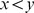 sequences, the outer loop concerns sectors for which 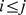. The inner loop iterates over all sequence subsets 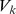 and performs on GPU relaxations of all matrices from 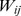 over all sequences from 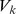. In order to perform the relaxation of particular 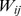 sector over 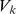, 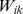 and 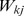 sectors are also required. As a result, for each calculated sector, a buffer for three sectors must be allocated at GPU. Sector sizes are adjusted in the way that buffer size does not exceed size of global memory 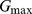.

**Figure 4:**
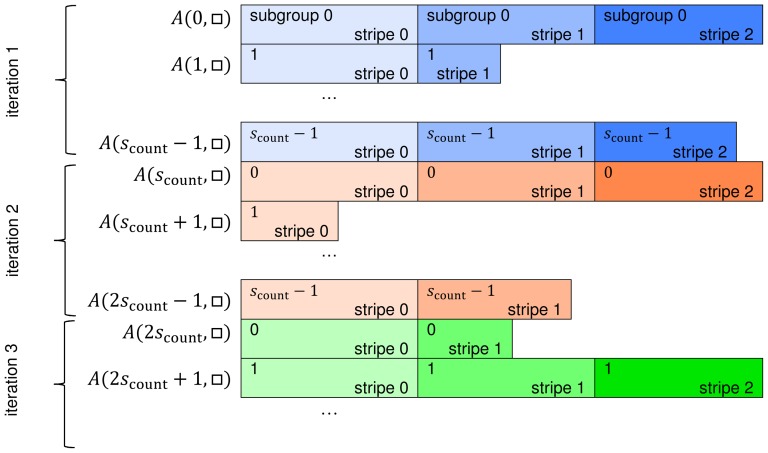
Scheme of traversing sparse matrix in multiplication procedure. 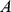
 There are 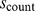 subgroups, each assigned to a single row of 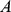 matrix. Each row is divided into stripes having 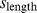 sparse elements. Subgroups process consecutive stripes of their rows. Stripes are represented by rectangles with corresponding subgroup identifier (upper left number) and their own identifier (bottom right number). Colours indicate stripes being calculated concurrently by a workgroup.

The relaxation procedure performed at GPU starts from weighting input matrix by 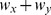 (sequence weights are computed during construction of the guide tree) and storing in 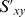. After that 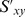 is relaxed over all sequences 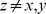. The relaxation over single sequence consists in performing a sparse-sparse matrix multiplication and adding the result to 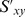. Let 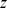 be the sequence over which 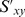 is relaxed. For simplicity let 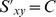, 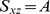, and 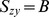. The update is performed as follows: 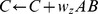. The multiplication is done by traversing all elements of 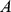, loading corresponding fragments of 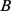 for each 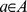, making computations and adding result to 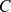. Processing of 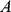 matrix is performed in horizontal blocks called *stripes*. There are 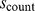 stripes executed concurrently, each having 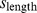 sparse elements. Let *subgroup* indicate a set of work-items which processes a stripe. As single sparse element is analysed by a single work-item, there are 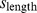 work-items in a subgroup and  work-items in a workgroup. An important note is that consecutive subgroups are assigned to consecutive rows of 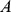 matrix, thus at any given moment only one stripe in a row is being processed. The scheme of traversing 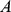 matrix is presented in Figure 4.

A detailed description of actions performed by a single subgroup is presented below. For convenience, let us introduce an additional notion: 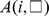
—
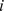
'th row of 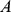, 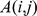
—
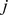
'th element of 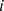
'th row of 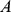, 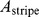
—some stripe of 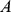, 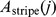
—
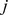
'th element of 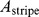. At the beginning, each work-item computes an identifier of its subgroup 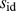 and an offset within it 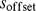. Then the subgroup sets itself on 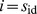 row and copies the first stripe 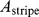 of 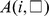 to local memory (each sparse element is copied by a single work-item). After that it iterates over sparse elements 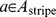. Note, that for each element 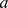 there is a corresponding row 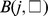 in 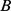 such that 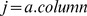. In the following steps, the subgroup processes consecutive stripes of these rows. Each work-item reads its sparse element 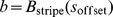 of the current stripe, calculates 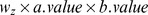 and adds it to the proper element of 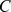. In MSAProbs 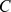 was temporarily stored in the dense form, therefore the algorithm updated directly 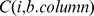. This approach is not used in QuickProbs as it renders inferior results. This is mainly because of increase in global memory requirements for a task which limits the size of a dataset that can be processed on a graphics processor. Moreover, as input matrices are sparse, accesses to 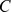 are non-coalesced which decreases performance. An alternative solution is to use local memory for buffering in the dense form only 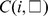 row. Nevertheless, as tests show, this variant is also inappropriate for graphics processor execution. Since maximum length of a dense row is 1024 and a single element is a 32-bit floating-point value, 8 dense rows are sufficient to fill whole 32 KB GPU local memory limiting GPU occupancy.

After considering all these factors, we decided to store 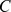 rows in local memory directly in the sparse form. The problem that emerges here concerns finding an element to be updated 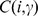, where 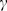 is index of a sparse element with column number equal to 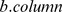. We examined two methods of overcoming this issue—hashing 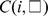 values according to column indices and performing binary search. As the latter turned out to be more efficient, it is employed in QuickProbs. If there is no index 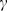 such that 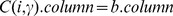, no update is performed (no new sparse elements can be introduced to 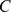). After subgroup finishes processing 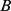 rows for all elements 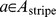, it reads another stripe of 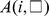. When processing of 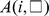 ends, 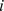 is incremented by 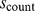 and the aforementioned scheme is repeated. After relaxing 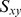 matrix over all sequences 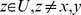, sparse elements smaller than 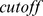 are filtered out. The pseudo-code of a single relaxation is presented in Table 4.

**Table 4:** Algorithm 4.

**Input:**	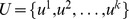 —set of sequences, 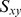 —sparse matrix to be relaxed, 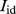 —work-item identifier, 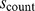 —number of stripes processed concurrently, 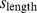 —stripe length.
**Output:**	Matrix 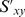 after relaxation.
1:	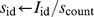
2:	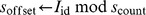
3:	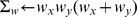
4:	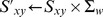
5:	**for all** 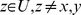 **do**
6:	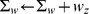
7:	relax ( 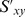 , 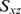 , 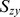 , 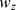 )
8:	**end for**
9:	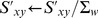
10:	**funtion** relax( 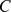 , 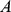 , 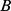 , 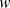 ) 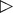 Function modifies C matrix
11:	**for** 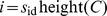 **do**
12:	**for all** 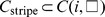 **do**
13:	Copy 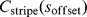 to local memory.
14:	**end for**
15:	synchronise
16:	**for all** 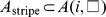 **do**
17:	Copy 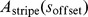 to local memory.
18:	synchronise
19:	**for all** 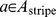 **do**
20:	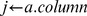
21:	**for all** 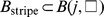 **do**
22:	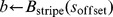
23:	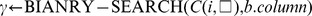
24:	**if** 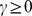 **then**
25:	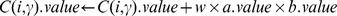
26:	**end if**
27:	**end for**
28:	**end for**
29:	synchronise
30:	**end for**
31:	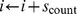
32:	**end for**
33:	**end function**

Pseudo-code of the posterior matrix relaxation procedure.

The relaxation of 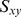 requires 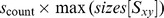 sparse elements for buffering 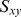 rows and 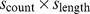 sparse elements for 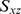 stripes. Additionally 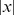 dense elements are needed for storing new sizes of 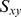 rows. Taking into account that a sparse element takes twice as much memory as a dense one, total local memory requirement for relaxation task equals 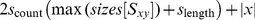
(8)


elements. In our preliminary experiments performed on both NVidia and AMD GPUs the best results were obtained for 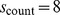 and 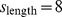. As we observed, for default value of 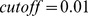, transforming matrix to sparse representation 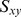 retains on average 5–10% of 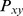 elements. Assuming that sequences are shorter than 1,000, which is often the case, 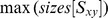 usually does not exceed 100. Local memory requirements are less than 12 KB/task assuming 32-bit elements. This results in better occupancy than in variant storing dense rows locally.

The most important disadvantage of the described method is its sensitivity for divergence of sparse matrices widths. This effect is especially visible for large datasets and is caused by multiplication algorithm containing several data-dependant nested loops. Branch divergence within a wavefront (warp) in outer-loop caused by different lengths of rows is very expensive, as alternative execution paths are time consuming. This effect is partially reduced by using stripes, but not entirely removed. To avoid waste of computational power, the diversity of sparse row lengths have to be minimised. The simplest way of obtaining it is gathering in sectors 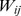 matrices of similar size. We plan to implement this feature in the next release of QuickProbs.

Final alignment construction and refinement. In MSAProbs the last stage of the algorithm is executed serially, which is appropriate for small datasets when profiles contain few sequences. When the number of sequences is larger, the computation time of stage IV becomes comparable to the execution times of stages I and III. Since QuickProbs uses significantly faster algorithms for stages I and III, the last stage, if performed serially, would be the most time consuming part of the entire algorithm. Thus, we decided to parallelise this stage for multi-core central processors. There are two main parallelisation approaches possible.

The first one follows inter-task concurrency scheme utilised by original MSAProbs for stages I and III. The Eqn. (3) is computed for all pairs of sequences 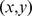 such that 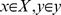. Processing of each pair can be treated as an independent task. In the assumed parallelisation model each thread executes one or several tasks. Second parallelisation scheme can be referred to as intra-task and consists in parallel computation of Eqn. (3). In theory it would be profitable even for small datasets. However, such computation model requires tens or hundreds times more threads than inter-task. Central processors, unlike GPUs, are not massively parallel devices, hence the costs of invocation and synchronisation of threads could be higher than the gains from parallel processing. This is why QuickProbs utilises inter-task parallelisation based on OpenMP as more appropriate for architecture of multi-core CPUs. In the future release we plan to customise stage IV for graphics processor execution. In this case, intra-task parallelisation will be the choice.

Beside parallelisation, QuickProbs introduces a simple optimisation to the final alignment calculation. When MSAProbs computes Eqn. (3), the processing is row-wise or column-wise, depending on the indexes of sequences 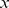 and 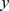, i.e., whether 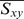 or 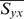 matrix is available. Since in stage III of QuickProbs both of these matrices are computed, the recurrence is always solved in a row-wise manner, which uses cache memory more effectively.

In QuickProbs the refinement stage is also done in parallel with the use of the profile alignment inter-task algorithm described above.

**Accurate mode.** QuickProbs, similarly to MSAProbs, has two parameters influencing quality of a final alignment, the number of consistency transformations 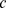 and the number of refinement iterations 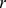. By default they equal 2 and 10, respectively. As QuickProbs in default settings turned out to be faster than its predecessor, we investigated whether it is possible to increase QuickProbs accuracy by tuning its parameters without exceeding MSAProbs computation times. At first we independently altered the number of refinement iterations to 20, 35, 50, 100, 150, 200 and the number of consistency transformations to 3, 4, 5. We established that increasing 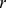 over default value 10 does not influence alignment accuracy, while increasing 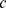 reduces results quality. This is because information loss caused by removing posterior elements below 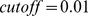 at the end of each transformation exceeds gains from the consistency. This was confirmed by the experiment in which 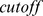 was set to 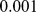 resulting in much less posterior elements being discarded and elevated accuracy. Increasing in this scenario number of consistency transformations to 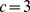 worsened accuracy. Even though, decrease is smaller than for 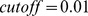, it is still noticeable suggesting that default 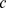 value produces best results.

The main disadvantage of approach based on global 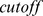 value is that fraction of elements removed from posterior matrices differs across sequence pairs. Thus, information loss for distant sequences (those having small 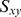 values) is much higher than for closely related sequences. Therefore, we decided to test an alternative variant in which 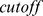 value is computed independently for each posterior matrix in order to retain assumed fraction 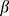 of elements. Such variant turned out to be a better compromise between computation time and result quality than static 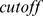. Thus, in the accurate mode of QuickProbs algorithm (referred to as QuickProbs-acc) we decided to use the adaptive filtering with 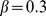 as it gave the best results in our experiments.

## Results

### Experimental setting

Experiments were performed on several hardware configurations built upon three CPUs (Intel Xeon W3550, Intel Xeon E5-2630, and AMD Phenom II X6 1090) and four graphics cards (NVidia GeForce GTX 480, 560, 680, and AMD Radeon HD 7970). All computers used for the tests were controlled by Windows operating system. Detailed parameters of hardware used in the experiments can be found in Table 5. As majority of modern computers are equipped with quad-core processors, the basic experimental platform contained Xeon W3550 CPU which was coupled with GeForce 480, 680, and Radeon 7970 GPUs. Another testing PC represented class of high-end desktop computers and consisted of Xeon E5-2630 hexa-core CPU and GeForce 680 which is one of the fastest single-chip graphic cards on the market. The experiments on quad-core PC reported Radeon 7970 to be faster than GeForce 680. However, AMD GPU was not supported properly by our hexa-core platform preventing us from testing it with Xeon E5-2630. Additional experiments were carried out on a mid-range hexa-core Phenom II X6 platform with GeForce 560. All examined processors are equipped with a dynamic overclocking feature which increases clocking frequency when CPU is not exceeding its thermal design power (this is usually the case when some cores are not loaded). Additionally, Intel processors utilise hyper-threading technology which improves task parallelism on a single core by duplicating some CPU logic. Operating system reports these processors to have twice as many cores as they physically posses.

**Table 5:** Characteristics of hardware used in the experiments.

			Cache					Local	
Hardware	Frequency	Cores	L1	L2	L3	Memory	Bandwidth	memory	GFLOPS
	[GHz]		[KB]	[KB]	[MB]	[GB]	[GB/s]	[KB]	
Xeon W3550	3.0–3.3	4^1^	4×32	4×256	8	24.0	26	—	50
Xeon E5-2630	2.3–2.8	6^1^	6×32	6×256	15	32.0	43	—	110
Phenom II X6	3.2–3.6	6	6×64	6×512	6	16.0	21	—	47
GeForce 480	1.4	15×32	15×16^2^	768	—	1.5	177	15×48^2^	1345
GeForce 560	1.8	8×48	8×16^2^	512	—	1.0	128	8×48^2^	1263
GeForce 680	1.0	8×192	8×16^2^	768	—	4.0	192	8×48^2^	3090
Radeon 7970	0.9	32×64	32×16	768	—	3.0	264	32×32	3789

Top three rows describe the CPUs. The bottom four rows describe the GPUs.

1 CPUs are equipped with HT technology. Table gives the number of physical cores.

2 On GeForce GPUs each compute unit posses 64 KB memory shared by L1 cache and local memory configured by default in ratio 16/48.

The main tests were performed on three popular benchmarks containing amino acid sequences, i.e., BAliBASE 3 [52], PREFAB 4 [9], and extended variant of OXBench [54]. All datasets were downloaded from Robert Edgar Web page in a standardised FASTA format [54]. All benchmarks are constituted of hundreds of sequence sets (see Table 6 for more details). As many algorithms were tuned on BAliBASE, sequence sets from this benchmark were shuffled randomly in order to remove potential bias. Additional experiments were carried out on real protein families downloaded from Pfam database [55]. The detailed characteristics of these datasets are presented in Table 7.

**Table 6:** Characteristics of amino-acid benchmarks used in experiments.

Dataset		Sequence statistics				No.
		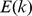	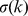	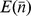	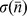	sets
BAliBASE	RV11	6.9	3.1	294	143	38
	RV12	9.0	5.8	389	256	44
	RV20	45.6	20.0	391	204	41
	RV30	63.2	34.1	359	155	30
	RV40	27.1	14.9	479	255	49
	RV50	27.9	13.8	488	138	16
BAliBASE all		28.5	26.2	396	219	218
PREFAB		45.2	10.8	289	126	1692
OXBench-X		122.5	100.7	147	82	395

A single set of sequences is described by the number of sequences 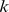 and the average sequence length 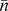.

Table presents means and standard deviations of these parameters for BAliBASE 3.0, PREFAB 4.0, and OXBench-X.

**Table 7:** Detailed characteristics of the protein families taken from Pfam database.

Dataset	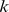	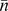	Description
PF02324	260	706	glycoside hydrolase
PF04762	404	667	IKI3 family
PF05110	483	611	AF4/FMR2-related protein
PF07095	600	642	growth attenuator protein
PF07520	283	878	virulence factor SrfB
PF08689	144	741	mediator complex subunit Med5
PF10136	317	626	site-specific recombinase
PF11573	204	794	mediator complex subunit 23

Each set is described by the number of sequences 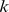 and the average sequence length 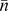.

For each dataset we calculated sum-of-pairs (SP) and column-scores (TC) measures with a use of Qscore software [56]. Note that PREFAB benchmark is constructed in the way that SP and TC are equal. All experiments were divided into two phases. First one consisted in comparing execution times of MSAProbs and QuickProbs stages across different hardware platforms. The results are presented in Tables 8, 9, 10, and 11. We report times for stage I (posterior matrix calculation), III (consistency transformation), IV (construction and refinement of final alignment). Stage II (building a guide tree) is omitted on purpose—its execution times are irrelevant with respect to the other stages and do not differ across hardware platforms. The overall times of algorithm execution are also given. Note, that for benchmark datasets tables present sum of processing times of all sequence sets within benchmarks. SP and TC scores were only used for checking the correctness of QuickProbs and are not reported. In Tables 8, 9, and 10 CPU results represent execution times of MSAProbs, while CPU+GPU configurations correspond to QuickProbs.

**Table 8:** Execution times for BAliBASE 3 benchmark reported in 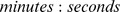 format.

Hardware	Stage I		Stage III		Stage IV		Total(I-IV)	
	time	speed-up	time	speed-up	time	speed-up	time	speed-up
Xeon W3550	39:33		5:08		9:13		53:54	
Xeon W3550 + GeForce 480	10:28	(×3.8)	2:43	(×2.2)	4:28	(×2.1)	17:21	(×3.1)
Xeon W3550 + GeForce 680	4:01	(×9.9)	2:12	(×2.3)	4:28	(×2.1)	10:42	(×5.0)
Xeon W3550 + Radeon 7970	2:58	(×13.3)	1:06	(×4.7)	4:27	(×2.1)	8:33	(×6.3)
Xeon E5-2630	30:35		2:45		10:05		43:31	
Xeon E5-2630 + GeForce 680	3:43	(×8.2)	2:10	(×1.3)	2:56	(×3.4)	8:51	(×4.9)
Phenom II X6	51:38		5:35		11:27		68:41	
Phenom II X6 + GeForce 560	12:21	(×2.9)	3:26	(×1.6)	3:24	(×3.3)	19:13	(×3.1)

Speed-ups of QuickProbs (CPU+GPU) over MSAProbs (CPU) across different hardware configurations are also shown.

**Table 9:** Execution times for PREFAB 4 benchmark reported in 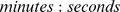 format.

Hardware	Stage I		Stage III		Stage IV		Total(I-IV)	
	time	speed-up	time	speed-up	time	speed-up	time	speed-up
Xeon W3550	169:19		16:11		23:15		208:50	
Xeon W3550 + GeForce 480	30:23	(×5.6)	9:15	(×1.8)	7:55	(×2.9)	47:40	(×4.4)
Xeon W3550 + GeForce 680	13:41	(×12.4)	8:46	(×1.8)	8:00	(×2.9)	30:34	(×6.8)
Xeon W3550 + Radeon 7970	8:47	(×19.2)	4:47	(×3.4)	7:55	(×2.9)	21:35	(×9.7)
Xeon E5-2630	130:50		9:39		23:59		164:36	
Xeon E5-2630 + GeForce 680	13:31	(×9.7)	8:42	(×1.1)	5:21	(×4.4)	27:42	(×5.9)
Phenom	231:41		18:34		31:53		282:16	
Phenom + GeForce 560	40:01	(×5.8)	13:47	(×1.4)	7:36	(×4.2)	61:29	(×4.6)

Speed-ups of QuickProbs (CPU+GPU) over MSAProbs (CPU) across different hardware configurations are also shown.

**Table 10:** Execution times for OXBench-X benchmark reported in 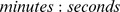 format.

Hardware	Stage I		Stage III		Stage IV		Total(I-IV)	
	time	speed-up	time	speed-up	time	speed-up	time	speed-up
Xeon W3550	133:49		89:12		17:36		240:58	
Xeon W3550 + GeForce 480	12:42	(×10.6)	77:59	(×1.1)	6:41	(×2.7)	98:02	(×2.5)
Xeon W3550 + GeForce 680	10:36	(×12.7)	71:28	(×1.2)	6:44	(×2.6)	89:26	(×2.7)
Xeon W3550 + Radeon 7970	7:19	(×18.3)	27:29	(×3.2)	6:36	(×2.7)	42:03	(×5.7)
Xeon E5-2630	101:03		62:55		18:09		182:41	
Xeon E5-2630 + GeForce 680	10:27	(×9.7)	70:57	(×0.9)	3:20	(×5.4)	85:28	(×2.1)
Phenom	184:03		123:25		24:35		332:23	
Phenom + GeForce 560	19:19	(×9.5)	110:50	(×1.1)	5:46	(×4.3)	136:04	(×2.4)

Speed-ups of QuickProbs (CPU+GPU) over MSAProbs (CPU) across different hardware configurations are also shown.

**Table 11:** Detailed results for the real-life datasets from Pfam database.

Dataset	MSAProbs				QuickProbs			
	I	III	IV	Total	I	III	IV	Total
PF02324	21:30	2:39	7:08	31:17	0:59(×22.0)	0:25(×6.3)	1:10(×6.1)	2:34(×12.2)
PF04762	46:54	13:37	16:45	77:18	1:54(× 24.7)	3:30(×3.9)	4:47(×3.5)	10:42(×7.2)
PF05110	55:36	23:13	18:34	97:27	12:22(×4.5)	6:27(×3.6)	5:20(×3.5)	25:32(×3.8)
PF07095	91:17	16:03	19:58	126:22	3:57(×23.0)	3:46(×4.3)	4:26(×4.3)	12:11(×10.3)
PF07520	39:40	5:01	14:07	58:52	1:42(×23.3)	0:39(×7.7)	2:29(×5.7)	4:53(×12.1)
PF08689	7:16	1:20	2:33	1:09	0:23(×18.7)	0:13(×6.3)	0:35(×4.4)	1:12(×9.3)
PF10136	25:19	4:24	4:42	34:26	1:10(×21.6)	1:05(×4.1)	1:14(×3.8)	3:37(×9.5)
PF11573	16:50	2:33	6:02	5:25	6:53(×2.4)	0:19(×8.0)	1:07(×5.4)	8:20(×3.0)
All	304:22	68:54	88:49	462:16	29:20(×10.4)	16:24(×4.2)	21:08(×4.2)	69:01(×6.7)

Times are given in 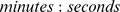 format.

The aim of the second experimental step was to compare base QuickProbs version and its accurate variant QuickProbs-acc with competing algorithms. SP and TC were used as quality measures and are reported together with total execution times in Table 12. Experiments were carried out on a machine equipped with Xeon W3550 and Radeon 7970. Packages that were chosen for experiments are MSAProbs 0.9.7, ClustalW v2.1, Clustal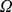 v1.2.0, Kalign2 v2.04, Kalign-LCS v1.0, MAFFT v7.053b, and MUSCLE v3.8.31. ClustalW was executed in the default and fast mode (distances calculated using full pairwise alignments and 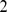-tuple matches, respectively). MAFFT was run in the default mode without consistency and auto mode which selectively turns consistency on. MSAProbs algorithm in both experimental parts was compiled with a use of MinGW compiler due to support of long double floating-point precision which is used in the implementation. Additionally, we examined MSA-CUDA, a ClustalW variant suited for graphics processors. As CUDA is not supported by AMD GPUs, MSA-CUDA was run on a platform with Xeon W3550 and GeForce 680.

**Table 12:** Qualitative results for BAliBASE, PREFAB, and OXBench-X run on Xeon W3550 + Radeon 7970 (GeForce 680 was used for MSA-CUDA).

Algorithm	BAliBASE			PREFAB		OXBench-X		
	time	SP	TC	time	SP/TC	time	SP	TC
QuickProbs-acc	28:19	87.9	60.8	64:48	74.0	255:36	89.3	80.2
MSAProbs	53:54	87.8	60.8	208:50	73.7	240:58	89.1	80.0
QuickProbs	8:33	87.8	60.7	21:35	73.6	42:03	89.1	80.0
MAFFT-auto	21:49	86.7	58.3	77:49	72.3	22:01	87.7	78.4
Clustal 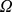	7:33	83.6	54.8	23:07	70.0	—	—	—
MUSCLE	15:17	81.7	46.8	40:38	67.7	30:36	87.5	77.6
Kalign-LCS	0:27	82.9	50.4	1:47	65.9	0:36	86.8	76.4
MAFTT-default	3:47	81.3	46.2	24:54	67.7	6:02	86.1	75.6
Kalign2	0:39	81.4	47.5	1:58	64.9	0:56	85.7	75.1
ClustalW	27:22	75.8	38.3	124:30	61.9	89:36	85.3	74.2
ClustalW-quicktree	6:25	73.7	37.1	22:19	61.9	8:04	84.9	73.8
MSA-CUDA	—	—	—	27:01	61.7	9:07	85.3	74.1
MSA-CUDA-quicktree	3:20	72.9	36.9	13:31	61.6	7:03	85.0	74.0

Aligners are sorted according to the average quality rank. Times are given in 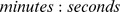 format.

### Benchmark datasets: central processor running times

The first observation when analysing results for benchmark datasets from Tables 8, 9, and 10 is that relative execution times of original MSAProbs agree with relative theoretical computational power of CPUs used in the experiments. Xeon E5-2630 is the fastest central processor and it outperforms Xeon W3550 and Phenom II X6, respectively second and third CPU in the comparison. Nevertheless, when analysing results of stage IV of MSAProbs which is implemented in serial manner, it is apparent that quad-core Xeon performs better than its hexa-core counterpart. This can be explained by the fact, that single core of Xeon W3550 has a higher clock rate and is faster than E5-2630 even though the latter represents a newer CPU generation. When stage IV is parallelised with a use of OpenMP, as it is done in QuickProbs, E5-2630 takes the first place. An interesting observation is, that Phenom performs in this situation better than Xeon W3550. We believe, this may be caused by the memory access pattern in this part of the algorithm that prefers AMD CPU cache architecture over competitor. Another important conclusion is that parallel variant of stage IV does not scale perfectly with the number of cores—speed-ups with respect to the serial version are always lower. This is probably because some parts of stage IV were not subject to an OpenMP parallelisation and run for the same time independently of the number of cores.

### Benchmark datasets: graphics processor running times

The shortest absolute execution times of analyses as well as highest speed-ups with respect to the original MSAProbs were obtained by Xeon W3550 + Radeon 7970 platform (see Tables 8, 9, 10). This coincides with a fact that Radeon has the greatest computational power from the examined GPUs. Since computations executed at graphic processor dominate in terms of execution time over CPU-parallel steps, this configuration is superior to the machine with faster CPU and slower GPU (E5-2630 + GeForce 680).

When detailed results for particular stages are analysed, the highest speed-ups are obtained for stage I. For Xeon W3550 + Radeon 7970 they vary from 13.3 (BAliBASE) to 19.2 (PREFAB). GeForce 680 turned out to be slower with speed-ups varying from 9.9 to 12.7. On hexa-core Xeon machine speed-ups are lower, but execution times are still superior to the original MSAProbs. Interesting observation comes from analysis of times obtained by GeForce 480 and 560, which are noticeably smaller than is suggested by the difference in computational power. After deeper investigation it became clear that the problem is caused by a number of registers in these GPUs which limits maximum size of a workgroup 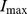 from 1024 to 576. Task can be processed at graphics processor if shorter of its sequence does not exceed 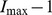. Therefore, smaller workgroups reduce performance as many tasks have to be calculated at CPU instead of GPU. Additionally, they limit occupancy for tasks executed at graphics processor preventing GPU computational power from full utilisation. In the case of stage III speed-ups of QuickProbs algorithm with respect to MSAProbs are lower than in stage I. For BAliBASE, PREFAB, and OXBench-X benchmarks run on quad-core CPU and Radeon 7970 they equal 4.7, 3.4, and 3.2, respectively. Significantly worse results were observed for GeForce 680 which indicates that parallelisation scheme of stage III prefers architecture of AMD GPUs. Another interesting observation is that differences between GeForce 480 cards and GeForce 680 are smaller than in the case of stage I. The explanation is that relaxation procedure is not limited by the register count. The worst speed-ups were reported for OXBench-X that contains large sets having hundreds of sequences. The consistency stage executed at GPU is vulnerable for divergence in width of sparse rows of input matrices. For large sets, the divergence is significant, thus lots of computational power is wasted resulting in lower speed-ups.

### Pfam datasets running times

Experiments on real protein families were performed on our fastest testing platform equipped with Xeon W3550 CPU and Radeon 7970 GPU. Results are presented in Table 11. As in benchmark datasets, the best speed-ups are observed for stage I—in the majority of cases they exceed 20 with a maximum value of 24.7. The only exceptions are PF05110 and PF11573 with speed-ups equal to 4.5 and 2.4, respectively. This is caused by the presence of long sequences (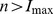) that are processed on the CPU and dominate whole posterior calculation stage. Speed-ups for stage III are smaller which also coincides with the benchmark results. They vary from 3.6 to 8.0. In the case of stage IV which was parallelised for multi-core CPUs, execution times are 3.5 to 6.1 times shorter than in MSAProbs. An interesting fact is that for the majority of datasets speed-ups exceed 4, the number of physical cores in our testing platform. This differs from benchmark results where speed-ups were always lower than number of cores. There are many reasons for this. Firstly, families from Pfam are much larger than benchmark sequence sets, thus proportions between parallel and serial operations are better. Thanks to this, the modification that improved cache utilisation is also more beneficial. Finally, Xeon W3550 CPU is equipped with a hyper-threading technology which increases task parallelism of a single core. The overall QuickProbs speed-up on all families equals 6.7. One must keep in mind, that this result is strongly skewed by PF05110 and PF11573 sets for which performance is limited by the presence of long sequences (stage I is the most time consuming part of the algorithm). If we exclude them from the comparison, the speed-up increases to 9.7.

### Comparison with other methods

Aligners from Table 12 were ranked on all datasets according to the result quality (SP and TC can be used interchangeably for this purpose as they generate identical ranks), and ordered by the average rank. The first group of algorithms gathers most accurate, consistency-based methods: QuickProbs in base and accurate variant, MSAProbs, and MAFFT-auto. The best aligner in terms of result quality is QuickProbs-acc which is superior to MSAProbs, the most accurate method so far. In order to statistically analyse observed differences in SP and TC, we performed Wilcoxon signed-rank tests [57] at the significance level 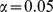. If one considers entire benchmarks, the significance was reported for PREFAB only (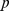-value = 0.000491). However, we suspected that adaptive filtering of sparse matrices used by QuickProbs-acc would be beneficial mainly for distantly related sequences, where static 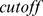 may result in the information loss. Therefore, we clustered sets in benchmarks according to the average sequence identity and analysed differences on groups. In the case of OXBench-X it turned out that for 94 sets from twilight zone (average identity below 30%), the predominance of QuickProbs-acc with respect to MSAProbs was statistically significant (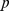-values equalled to 0.002074 and 0.024536 for SP and TC, respectively). Same analysis performed for PREFAB also revealed strong evidence that QuickProbs-acc is particularly suited for distantly related sequences: 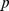-value for 535 twilight sets was two orders of magnitude lower than in the case of whole benchmark and equalled 0.000004. In the case of BAliBASE dataset, we compared different RV groups but no significant differences were discovered. An important observation is that all these results were obtained without exceeding MSAProbs execution times (in the case of BAliBASE and PREFAB QuickProbs-acc was much faster, for OXBench-X it was only 6% slower). This is important, since MSAProbs is the most time consuming from all tested algorithms.

In cases where MSAProbs accuracy is sufficient, default mode of QuickProbs should be used as it produces almost identical results as MSAProbs, at a fraction of the time. Small discrepancy in accuracies is caused by using double floating-point precision in partition function calculation at GPU instead of long double. This was confirmed by the fact, that MSAProbs compiled without long double support gives exactly the same results as QuickProbs. Fourth aligner in terms of quality rank was MAFFT in automatic mode. Additionally, it turned out to be slower than QuickProbs on BAliBASE and PREFAB benchmarks.

Second group of packages consists of algorithms without consistency: Clustal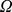, Kalign-LCS, MUSCLE, MAFTT-default, Kalign2, and ClustalW. The first from aforementioned methods, Clustal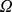 produces the best results on BAliBASE and PREFAB datasets, however, it failed to run properly on OXBench-X. The best algorithm which executed successfully on all datasets is MUSCLE. Its execution times may be an issue in some applications, though. Kalign-LCS, the modification of Kalign2, is inferior to MUSCLE only by a small margin. Moreover, it is the fastest method in the comparison outperforming also CUDA-based algorithms, which makes it the best choice when one is interested in aligning large sets of sequences. An interesting observation is that ClustalW performs significantly worse than other aligners in comparison. Taking into account relatively long execution times, it is clear that ClustalW, still the most popular MSA software, should be replaced in biological analyses by other packages. The ClustalW variant suited for GPU processing (MSA-CUDA) is characterised by much shorter execution times. Nevertheless, it is still inferior to fast and more accurate CPU aligners like Kalign-LCS. Additionally, its default variant failed to execute properly on BAliBASE benchmark.

## Discussion

In the paper we present QuickProbs, a variant of MSAProbs algorithm suited for graphics processors. We designed and implemented GPU versions of two most time consuming stages of the strategy, which originally were customised for multi-core architecture with the use of OpenMP. These are the calculation of posterior probability matrices and the consistency transformation. Posterior matrices are calculated on the basis of pair hidden Markov models and partition functions. From algorithmic point of view the stage performs several dynamic programming passes. Customising computation scheme to massively parallel GPU architecture, optimising global memory accesses by using jagged pattern and exploiting advanced method of work balancing resulted in significant speed-ups at this stage. On the main testing platform equipped with a quad-core Xeon W3550 and Radeon 7970, QuickProbs calculated posterior matrices as much as 24.7 times faster than original CPU-parallel method. The consistency transformation stage relies on performing set of small sparse matrix multiplications. We designed an algorithm for this purpose customised for graphics processors. As experiments on the basic testing platform show, it outperforms its MSAProbs equivalent with speed-ups reaching 8.0. In order to further improve execution times, we additionally suited the last stage of QuickProbs for multi-core CPU architectures with a use of OpenMP. Thanks to this, the construction and refinement of final alignment is done even 6.1 times faster than previously.

Assessed on BAliBASE, PREFAB, and OXBench-X benchmarks, QuickProbs turned out to be respectively, 6.3, 9.7, and 5.7 times faster than MSAProbs. In the experiments on protein families from Pfam database, the overall speed-up was 6.7. This makes QuickProbs competitive to faster aligners like MAFFT, Clustal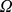, or MUSCLE. In the research we additionally introduced QuickProbs-acc, a tuned version of QuickProbs which is significantly more accurate than MSAProbs on sequence sets from twilight zone (identity 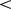 30%) without exceeding its running time. Unlike previously published GPU-suited multiple sequence alignment methods, computations in QuickProbs algorithm are performed in OpenCL making it suitable for graphics processors produced by both main vendors, NVidia and AMD.

Our future plans focuses three main tasks: (1) removing limitation of sequence lengths that can be processed at GPU at posterior stage, (2) redesigning consistency transformation to make it less vulnerable for divergence in sparse rows lengths, (3) customising final alignment construction and refinement procedures for graphics processors.

QuickProbs algorithm together with all the datasets used in the research are available at http://adaa.polsl.pl/agudys/quickprobs/quickprobs.htm. The detailed analysis of the time complexity of MSAProbs and QuickProbs can be found in the Supplement S1.

## Supporting Information

Supplement S1 **Detailed analysis of MSAProbs and QuickProbs time complexities.**
(PDF)

## References

[1] Wang L, Jiang T (1994) On the complexity of multiple sequence alignment. Journal of Computational Biology 1: 337–348. 8790475 10.1089/cmb.1994.1.337

[2] Just W (1999) Computational complexity of multiple sequence alignment with SP-Score. Journal of Computational Biology 8: 615–623. 10.1089/106652701753307511 11747615

[3] Feng DF, Doolittle RF (1987) Progressive sequence alignment as a prerequisite to correct phylogenetic trees. Journal of Molecular Evolution 25: 351–360. 3118049 10.1007/BF02603120

[4] Barton GJ, Sternberg MJ (1987) A strategy for the rapid multiple alignment of protein sequences. Confidence levels from tertiary structure comparisons. Journal of Molecular Biology 198: 327–337. 3430611 10.1016/0022-2836(87)90316-0

[5] Krogh A, Brown M, Mian IS, Sjölander K, Haussler D (1994) Hidden Markov models in computational biology: applications to protein modeling. Journal of Molecular Biology 235: 1501–1531. 8107089 10.1006/jmbi.1994.1104

[6] Thompson JD, Higgins DG, Gibson TJ (1994) CLUSTAL W: improving the sensitivity of progressive multiple sequence alignment through sequence weighting, position-specific gap penalties and weight matrix choice. Nucleic Acids Research 22: 4673–4680. 7984417 10.1093/nar/22.22.4673 PMC308517

[7] Notredame C, Higgins D, Heringa J (2000) T-Coffee: A novel method for fast and accurate multiple sequence alignment. Journal of Molecular Biology 302: 205–217. 10964570 10.1006/jmbi.2000.4042

[8] Katoh K, Misawa K, Kuma K, Miyata T (2002) MAFFT: a novel method for rapid multiple sequence alignment based on fast Fourier transform. Nucleic Acids Research 30: 3059–3066. 12136088 10.1093/nar/gkf436 PMC135756

[9] Edgar RC (2004) MUSCLE: multiple sequence alignment with high accuracy and high throughput. Nucleic Acids Research 32: 1792–1797. 15034147 10.1093/nar/gkh340 PMC390337

[10] Do C, Mahabhashyam M, Brudno M, Batzoglou S (2005) ProbCons: Probabilistic consistencybased multiple sequence alignment. Genome Research 15: 330–340. 15687296 10.1101/gr.2821705 PMC546535

[11] Roshan U, Livesay DR (2006) Probalign: multiple sequence alignment using partition function posterior probabilities. Bioinformatics 22: 2715–2721. 16954142 10.1093/bioinformatics/btl472

[12] Liu Y, Schmidt B, Maskell D (2010) MSAProbs: multiple sequence alignment based on pair hidden Markov models and partition function posterior probabilities. Bioinformatics 26: 1958–1964. 20576627 10.1093/bioinformatics/btq338

[13] O′Sullivan O, Suhre K, Abergel C, Higgins D, Notredame C (2004) 3DCoffee: Combining protein sequences and structures within multiple sequence alignments. Journal of Molecular Biology 340: 385–395. 15201059 10.1016/j.jmb.2004.04.058

[14] Deng X, Cheng J (2011) MSACompro: protein multiple sequence alignment using predicted secondary structure, solvent accessibility, and residue-residue contacts. BMC Bioinformatics 12: 472. 22168237 10.1186/1471-2105-12-472 PMC3299741

[15] Katoh K, Kuma Ki, Toh H, Miyata T (2005) MAFFT version 5: improvement in accuracy of multiple sequence alignment. Nucleic Acids Research 33: 511–518. 15661851 10.1093/nar/gki198 PMC548345

[16] Huerta-Cepas J, Capella-Gutierrez S, Pryszcz LP, Denisov I, Kormes D, et al (2011) PhylomeDB v3.0: an expanding repository of genome-wide collections of trees, alignments and phylogeny-based orthology and paralogy predictions. Nucleic Acids Research 39: 556–560. 21075798 10.1093/nar/gkq1109 PMC3013701

[17] Capella-Gutierrez S (2012) Analysis of multiple protein sequence alignments and phylogenetic trees in the context of phylogenomics studies. Pompeu Fabra UniversityPh.D. thesis

[18] Lassmann T, Sonnhammer E (2005) Kalign|an accurate and fast multiple sequence alignment algorithm. BMC Bioinformatics 6: 298. 16343337 10.1186/1471-2105-6-298 PMC1325270

[19] Lassmann T, Frings O, Sonnhammer E (2009) Kalign2: high-performance multiple alignment of protein and nucleotide sequences allowing external features. Nucleic Acids Research 37: 858–865. 19103665 10.1093/nar/gkn1006 PMC2647288

[20] Wu S, Manber U (1992) Fast text searching: allowing errors. Communications of the ACM 35: 83–91.

[21] Muth R, Manber U (1996) Approximate multiple string search. In: Proceedings of the 7th Annual Symposium on Combinatorial Pattern Matching. pp. 75-86.

[22] Deorowicz S, Debudaj-Grabysz A, Gudyś A (2014) Kalign-LCS|more accurate and faster variant of Kalign2 algorithm for the multiple sequence alignment problem. In: Man-Machine Interactions 3, Springer Cham Heidelberg New York Dordrecht London. pp. 495-502.

[23] Katoh K, Toh H (2007) Parttree: an algorithm to build an approximate tree from a large number of unaligned sequences. Bioinformatics 23: 372–374. 17118958 10.1093/bioinformatics/btl592

[24] Sievers F, Wilm A, Dineen D, Gibson T, Karplus K, et al (2011) Fast, scalable generation of highquality protein multiple sequence alignments using Clustal Omega. Molecular Systems Biology 7: 539. 21988835 10.1038/msb.2011.75 PMC3261699

[25] Blackshields G, Sievers F, Shi W, Wilm A, Higgins D (2010) Sequence embedding for fast construction of guide trees for multiple sequence alignment. Algorithms for Molecular Biology 5: 21. 20470396 10.1186/1748-7188-5-21 PMC2893182

[26] Liu W, Schmidt B, Voss G, Muller-Wittig W (2006) GPU-ClustalW: Using graphics hardware to accelerate multiple sequence alignment. Lecture Notes in Computer Science 4297: 363–374.

[27] Liu Y, Schmidt B, Maskell D (2009) MSA-CUDA: Multiple sequence alignment on graphics processing units with CUDA. In: Proceedings of the 20th IEEE International Conference on Applicationspecific Systems, Architectures and Processors. pp. 121-128.

[28] Gudyś A, Deorowicz S (2012) A parallel algorithm for the constrained multiple sequence alignment problem designed for GPUs. International Journal of Foundations of Computer Science 23: 877–901.

[29] Lin YS, Lin CY, Li ST, Lee JY, Tang CY (2010) GPU-REMuSiC: the implementation of constrain multiple sequence alignment on graphics processing units. In: Proceedings of the 2010 GPU Technology Conference. NVidia.

[30] Blazewicz J, Frohmberg W, Kierzynka M, Wojciechowski P (2013) G-MSA|A GPU-based, fast and accurate algorithm for multiple sequence alignment. Journal of Parallel and Distributed Computing 73: 32–41.

[31] OpenMP ARB (2013) OpenMP Application Program Interface version 4.0. Available: http://www.openmp.org/mp-documents/OpenMP4.0.0.pdf.

[32] Manavski S, Valle G (2008) CUDA compatible GPU cards as efficient hardware accelerators for Smith-Waterman sequence alignment. BMC Bioinformatics 9: S10. 10.1186/1471-2105-9-S2-S10 PMC2323659 18387198

[33] Ligowski L, Rudnicki W (2009) An efficient implementation of Smith Waterman algorithm on GPU using CUDA, for massively parallel scanning of sequence databases. In: Proceedings of the 2009 IEEE International Symposium on Parallel&Distributed Processing. Washington,USA: IEEE Computer Society, pp. 1-8.

[34] Liu Y, Schmidt B, Maskell D (2010) CUDASW++2.0: enhanced Smith-Waterman protein database search on CUDA-enabled GPUs based on SIMT and virtualized SIMD abstractions. BMC Research Notes 3: 93. 20370891 10.1186/1756-0500-3-93 PMC2907862

[35] Khajeh-Saeed A, Poole S, Perot J (2010) Acceleration of the Smith-Waterman algorithm using single and multiple graphics processors. Journal of Computational Physics 229: 4247–4258.

[36] Blazewicz J, Frohmberg W, Kierzynka M, Pesch E, Wojciechowski P (2011) Protein alignment algorithms with an efficient backtracking routine on multiple GPUs. BMC Bioinformatics 12: 181. 21599912 10.1186/1471-2105-12-181 PMC3125261

[37] Liu Y, Wirawan A, Schmidt B (2013) CUDASW++ 3.0: accelerating Smith-Waterman protein database search by coupling CPU and GPU SIMD instructions. BMC Bioinformatics 14: 117. 23557111 10.1186/1471-2105-14-117 PMC3637623

[38] Liu CM, Wong T, Wu E, Luo R, Yiu SM, et al (2012) SOAP3: ultra-fast GPU-based parallel alignment tool for short reads. Bioinformatics 28: 878–879. 22285832 10.1093/bioinformatics/bts061

[39] Chang DJ, Kimmer C, Ouyang M (2010) Accelerating the Nussinov RNA folding algorithm with CUDA/GPU. In: Proceedings of the 10th IEEE International Symposium on Signal Processing and Information. IEEE Computer Society, pp. 120-125: 20.

[40] Suchard MA, Rambaut A (2009) Many-core algorithms for statistical phylogenetics. Bioinformatics 25: 1370–1376. 19369496 10.1093/bioinformatics/btp244 PMC2682525

[41] Demouth J (2012) Sparse Matrix-Matrix Multiplication on the GPU. In: Proceedings of the GPU Technology Conference 2012. NVidia.

[42] NVidia (2013) CUSP library version 0.4.0. Available: https://developer.nvidia.com/cusp.

[43] NVidia (2013) cuSPARSE library version 5.5. Available: https://developer.nvidia.com/cusparse.

[44] Durbin R, Eddy SR, Krogh A, Mitchison G (1998) Biological Sequence Analysis: Probabilistic Models of Proteins and Nucleic Acids. Cambridge University Press.

[45] Thompson JD, Plewniak F, Poch O (1999) A comprehensive comparison of multiple sequence alignment programs. Nucleic Acids Research 27: 2682–2690. 10373585 10.1093/nar/27.13.2682 PMC148477

[46] Stoye J, Evers D, Meyer F (1998) Rose: generating sequence families. Bioinformatics 14: 157–163. 9545448 10.1093/bioinformatics/14.2.157

[47] NVidia (2013) CUDA Parallel Computing Platform version 5.5. Available: http://docs.nvidia.com/cuda/pdf/CUDA_C_Programming_Guide.pdf.

[48] Khronos Group (2013) The OpenCL Specification version 2.0. Available: http://www.khronos.org/registry/cl/specs/opencl-2.0.pdf.

[49] Viterbi A (1967) Error bounds for convolutional codes and an asymptotically optimum decoding algorithm. IEEE Transactions on Information Theory 13: 260–269.

[50] Sneath P, Sokal R (1973) Numerical Taxonomy. The Principles and Practice of Numerical Classification. San Francisco, USA: W.H. Freeman Limited.

[51] Needleman S, Wunsch C (1970) A general method applicable to the search for similarities in the amino acid sequence of two proteins. Journal of Molecular Biology 48: 443 - 453. 10.1016/0022-2836(70)90057-4 5420325

[52] Thompson J, Koehl P, Ripp R, Poch O (2005) BAliBASE 3.0: latest developments of the multiple sequence alignment benchmark. Proteins 61: 127–136. 16044462 10.1002/prot.20527

[53] Raghava GPS, Searle S, Audley P, Barber J, Barton G (2003) OXBench: A benchmark for evaluation of protein multiple sequence alignment accuracy. BMC Bioinformatics 4: 47. 14552658 10.1186/1471-2105-4-47 PMC280650

[54] Edgar RC (2009) Benchmark collection. Available: http://www.drive5.com/bench.

[55] Finn RD, Tate J, Mistry J, Coggill PC, Sammut SJ, et al (2008) The Pfam protein families database. Nucleic Acids Research 36: D281–D288. 18039703 10.1093/nar/gkm960 PMC2238907

[56] Edgar RC (2009) QSCORE multiple alignment scoring software. Available: http://www.drive5.com/qscore.

[57] Wilcoxon F (1945) Individual Comparisons by Ranking Methods. Biometrics Bulletin 1: 80–83.

