# Correction: QuickProbs—A Fast Multiple Sequence Alignment Algorithm Designed for Graphics Processors

**DOI:** 10.1371/journal.pone.0103051

**Published:** 2014-07-23

**Authors:** The PLOS ONE Staff

In the Materials and Methods section, under the subsection titled “QuickProbs algorithm,” there is an error in Equation 6. Please view the complete, correct equation here:



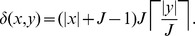



In Table 3 (Algorithm 3) the word “to” is missing in the “for” instruction in line 14. Please see the corrected Table 3 here:

**Table 3:** Algorithm 3.

**Input: ** 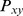 —dense matrix to be translated to sparse form, 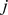 —work-item identifier (column number)
**Output**: 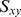 —output sparse matrix described by 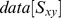 , 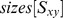 and 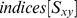 arrays.
1: Initialise in parallel 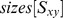 vector with 0.
2: synchronise
3: **for ** 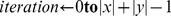 **do ** 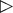 First pass: fill sizes vector.
4: 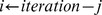
5: **if ** 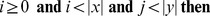
6: **if ** 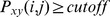 **then**
7: 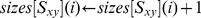
8: **end if**
9: **end if**
10: synchronise
11: **end for**

12: **if ** 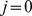 ** then**
13: 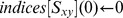
14: **for ** 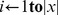 **do**
15: 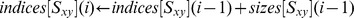
16: **end for**
17: **end if**
18: synchronise
19: Copy in parallel 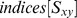 to auxiliary vector 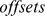 .
20: synchronise

21: **for** 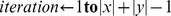 **do ** 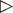 Second pass: fill sparse elements array.
22: 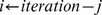
23: **if ** 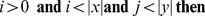
24: **if** 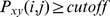 **then**
25: 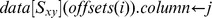
26: 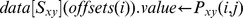
27: 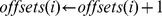
28: **end if**
29: **end if**
30: synchronise
31: **end for**

Pseudo-code of the sparse matrix generation procedure.

In Table 4 (Algorithm 4) the word “to” is missing in the “for” instruction in line 11. Please see the corrected Table 4 here:

**Table 4:** Algorithm 4.

**Input: ** 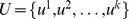 —set of sequences, 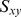 —sparse matrix to be relaxed, 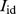 —work-item identifier, 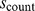 —number of stripes processed concurrently, 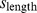 —stripe length.
**Output:** Matrix 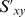 after relaxation.
1: 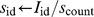
2: 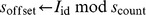
3: 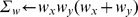
4: 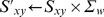
5: **for all ** 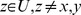 **do**
6: 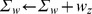
7: relax ( 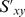 , 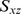 , 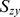 , 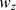 )
8: **end for**
9: 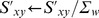
10: **funtion** relax( 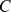 , 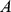 , 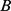 , 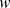 ) 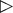 Function modifies C matrix
11: **for** 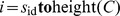 **do**
12: **for all** 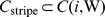 **do**
13: Copy 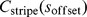 to local memory.
14: **End for**
15: synchronise

16: **for all ** 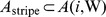 do
17: Copy 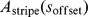 to local memory.
18: synchronise
19: **for all ** 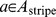 **do**
20: 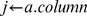
21: **for all** 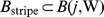 **do**
22: 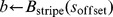
23: 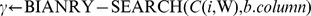
24: **if ** 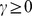 **then**
25: 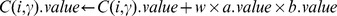
26: **end if**
27: **end for**
28: **end for**
29: synchronise
30: **end for**
31: 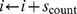
32: **end for**
33: **end function**

Pseudo-code of the posterior matrix relaxation procedure.

## References

[1] Gudyś A, Deorowicz S (2014) QuickProbs—A Fast Multiple Sequence Alignment Algorithm Designed for Graphics Processors. PLoS ONE 9(2): e88901 doi:10.1371/journal.pone.0088901 24586435 10.1371/journal.pone.0088901 PMC3934876

