# Supplementary material for: QuickProbs—A Fast Multiple Sequence Alignment Algorithm Designed for Graphics Processors
Source: PLoS One. 2014 Feb 25;9(2):e88901. doi: 10.1371/journal.pone.0088901 (PMC3934876; doi:10.1371/journal.pone.0088901)
Supplement: Supplement S1 — Detailed analysis of MSAProbs and QuickProbs time complexities. (PDF) [file pone.0088901.s001.pdf]

QuickProbs—a fast multiple sequence alignment algorithm designed for graphics processors. The complexity analysis.

Adam Gudyś[1,*], Sebastian Deorowicz[1]
1 Institute of Informatics, Faculty of Automatic Control, Electronics and Computer Science, Silesian University of Technology, Gliwice, Poland
∗ E-mail: adam.gudys@polsl.pl

1 Definitions and assumptions

In the following supplement we carry out a detailed analysis of QuickProbs time complexity. For convenience we also consider the complexity of original (CPU-parallel) MSAProbs algorithm as well as its serial variant. The latter will be denoted as MSAProbs-seq and is only a hypothetical algorithm (we discarded operations made by MSAProbs which are needless for serial execution).

Let us assume that $U = \left\{u^1, u^2, \ldots, u^k\right\}$ is the set of k input sequences of length n. The sequences in U can be ordered according to some relation '$<$'. Posterior matrix for two selected sequences $x, y \in U$ will be denoted as P_{xy} and S_{xy} for dense and sparse representations, respectively. Elements in the sparse representation are pairs consisting of *column* and *value*. In MSAProbs and basic variant of QuickProbs S_{xy} is produced by filtering from P_{xy} elements smaller than some assumed *cutoff* value. Such approach is however difficult for analysis as a number of elements in resulting sparse matrices is data dependant. Therefore, we assume that fraction of posterior matrix elements retained when transforming from the dense to the sparse representation is a constant β. Therefore, if P_{xy} matrix has size $(n+1) \times (n+1)$, its sparse representation S_{xy} contains $\beta(n+1)^2$ elements. This corresponds exactly to QuickProbs-acc algorithm variant described in the main paper. As experiments on MSAProbs and basic QuickProbs show, in the majority of cases a fraction of retained elements is between 0.05 and 0.1. Thus, the complexity analysis for constant β carried out in this supplement can be used as an approximation for filtering based on static *cutoff* which is employed by MSAProbs and basic QuickProbs.

Let us introduce a notion describing hardware platform on which the algorithm is executed:

- p—number of cores in central processor,
- q_1—number of compute units in graphics processor,
- q_2—number of processing elements per compute unit in graphics processor,
- w—wavefront/warp size (smallest number of work-items executed physically in parallel at graphics processor). Number of processing elements q_2 is a multiplicity of w.

The definitions of OpenCL terms used above can be found in the main paper.

In the analysis we assume some relations between dataset size and hardware architecture. They concern number of sequences k and sequence lengths n and hold for majority of datasets beside trivial cases containing very few or very short sequences. These are:

Assumption 1: $k(k-1)/2 > p$,

Assumption 2: $k(k-1)/2 > q_1$,

Assumption 3: $n > w$,

Assumption 4: $n > p$.

An additional assumption concerns relation between coefficient β and sequence length n:

Assumption 5: $\beta > \frac{1}{n}$.

Time complexities are denoted as $T(k,n)$. The name of the procedure or stage number it refers to is given in a superscript. In the case of QuickProbs algorithm, all operations are divided into three categories: graphics processor computations, central processor computations and CPU-GPU memory transfers. The corresponding category names (CPU, GPU and MEM) are given in a subscript. E.g., $T(k,n)^{\mathrm{I}}_{\mathrm{GPU}}$ indicates the time complexity of GPU operations performed in stage I of QuickProbs algorithm.

In order to simplify formulations in the supplement we introduce an additional notion concerning rounding of numbers. Namely, $\lceil x \rceil_y$ denotes rounding x up to the closest multiplicity of y and is defined as $\left\lceil \frac{x}{y} \right\rceil y$.

2 Serial algorithm

2.1 Posterior matrix calculation

Stage I of the algorithm consists in calculating dense posterior probability matrices P_{xy} and pairwise distances d_{xy} for all $x, y \in U, x < y$. After that P_{xy} matrices are transformed to sparse representations S_{xy}. The procedure of calculating particular P_{xy}, d_{xy} and S_{xy} is called a posterior task. There are $k(k-1)/2$ posterior tasks processed sequentially by MSAProbs-seq algorithm.

In order to calculate particular P_{xy} and d_{xy}, the algorithm performs several dynamic programming passes over matrices of size $(n+1) \times (n+1)$. After that it selects from P_{xy} matrix β greatest elements to produce S_{xy}. Execution of a single posterior task requires $\Theta(n^2)$ operations. The procedure is repeated for all tasks. As a result the time complexity of stage I of MSAProbs-seq does not depend on the data and is

$$T^{\mathrm{I}}(k,n) = \Theta\left(k^2 n^2\right). \quad (1)$$

2.2 Tree construction and sequence weighting

The guide tree construction in MSAProbs-seq is performed with a use of UPGMA algorithm. After that sequences are weighted in order described by the tree using ClustalW weighting scheme. The worst and average case time complexity of these operations is

$$T^{\mathrm{II}}(k) = \Theta(k^2). \quad (2)$$

The weight of sequence $u \in U$ will be referred to as w_u.

2.3 Consistency transformation

The consistency transformation stage produces S'_{xy} matrices by performing relaxation of $S_{xy}, x < y$ matrices over all sequences $z \in U, z \neq x, y$. The calculation of given S'_{xy} will be referred to as a relaxation task. It starts from weighting S_{xy} elements by $w_x + w_y$ and storing result in S'_{xy}. The relaxation of S_{xy} over sequence z consists in performing multiplication of S_{xz} and S_{zy} sparse matrices, weighting the result by w_z and adding to S'_{xy}. In MSAProbs-seq algorithm the S'_{xy} matrix is temporarily transformed to the dense form P'_{xy}. For simplicity let $P'_{xy} = C$, $S_{xz} = A$, and $S_{zy} = B$. Additionally, $A(i, \square)$ denotes i'th row of A. The relaxation is done as follows: $C \leftarrow C + w_z AB$. The procedure iterates over consecutive $A(i, \square)$ rows. For each element $a \in A(i, \square)$ it accesses a row $B(j, \square)$ such that $j = a.column$. Then it iterates over $b \in B(j, \square)$ elements and adds $w_z \times a.value \times b.value$ to $C(i, b.column)$.

There are βn^2 elements in A. The number of elements in B rows accessed during the procedure depends on the data. It is maximal when A contains n non-empty rows of length βn while B contains βn non-empty rows of length n. Moreover, A rows have to be identical and their elements have to point

to all non-empty B rows. As the number of accesses to B is crucial for running time of the relaxation procedure, the above-mentioned case is also the worst case. Its time complexity is

$$T^{\text{relax-worst}}(n) = \Theta(\beta n^3). \tag{3}$$

The worst case is however very unlikely to happen for the real data. For practical applications, the knowledge about average complexity of the procedure would be more convenient. The problem is that such analysis requires distribution of sparse rows lengths in posterior matrices which is unknown. Therefore we decided to concern a special case where all sparse rows are of the same length βn. Such situation is also unrealistic for the real-life alignment problems, though it is more similar to them than the worst case. In the above-mentioned conditions single relaxation requires

$$T^{\text{relax-special}}(n) = \Theta(\beta^2 n^3) \tag{4}$$

operations.

A single task consist of $k-2$ multiplications. As there are c consistency transformations in MSAProbs-seq each executing $k(k-1)/2$ tasks, the time complexity of the whole stage III is

$$T^{\text{III}}(k,n) = \Theta(ck^3 T^{\text{relax}}(n)). \tag{5}$$

For the worst case it gives

$$T^{\text{III-worst}}(k,n) = \Theta(c\beta k^3 n^3), \tag{6}$$

and for the special case of equally long sparse rows

$$T^{\text{III-special}}(k,n) = \Theta(c\beta^2 k^3 n^3). \tag{7}$$

2.4 Final alignment construction and refinement

The final alignment is constructed progressively, according to the order described by the guide tree. At each progressive step QuickProbs aligns two sets of aligned sequences called profiles. Let X and Y be the profiles of lengths n_1 and n_2 containing k_1 and k_2 sequences, respectively. The first step is to compute posterior matrix P_{XY} for X and Y. This matrix is stored in the dense form, thus its size is $n_1 n_2$. The procedure is done by accumulating in P_{XY} elements of all pairwise posterior matrices S_{xy} such that $x \in X$ and $y \in Y$. As each S_{xy} matrix contains βn^2 elements, this is done in $\Theta(\beta k_1 k_2 n^2 + n_1 n_2)$. After that, a dynamic programming is performed over P_{XY} in order to calculate profile-profile alignment. This requires additional $n_1 n_2$ operations. Eventually, the time complexity of the procedure is

$$T^{\text{profile}}(k_1, k_2, n_1, n_2, n) = \Theta\left(\beta k_1 k_2 n^2 + n_1 n_2\right). \tag{8}$$

Note, that the length of a profile containing k_1 sequences is at least n (when all sequences within profile are perfectly aligned) and at most $k_1 n$ (when all sequences within profile are misaligned). In the general case, we can express the profile length as nk_1^{ϕ}, where ϕ is some value from $\langle 0, 1\rangle$ interval. This parameter can be treated as a value of a function describing similarity of sequences in a profile. We can use this function to reformulate the time complexity of the profile-profile alignment as

$$T^{\text{profile}}(k_1, k_2, n) = \Theta\left(n^2\left(\beta k_1 k_2 + k_1^{\phi} k_2^{\phi}\right)\right). \tag{9}$$

The important issue when determining complexity of the whole procedure is that the number of progressive steps as well as sizes of profiles to be aligned at each step depend on the tree structure. In our analysis we consider two extreme cases with perfectly balanced (1) and perfectly unbalanced (2) trees. For simplicity let us assume that the total number of sequences is $k = 2^L$, for some positive integer L, and that function ϕ is constant for all profiles.

The height of a perfectly balanced tree (case 1) is $\text{loq}_2 k + 1$. Progressive steps are performed on all levels beside the bottom one. Thus, only $L = \text{loq}_2 k$ top levels are of our interest. At the i'th level of the tree (the top is assumed to be level 1) there are 2^{i-1} nodes, each representing a profile-profile alignment. Input profiles at i'th level contain 2^{L-i} sequences, each. The time complexity of the whole procedure is then

$$\begin{aligned}
&\Theta\left(\sum_{i=1}^{L} 2^{i-1}\; T^{\text{profile}}(2^{L-i}, 2^{L-i}, n)\right) = \\
&= \Theta\left(\sum_{i=1}^{L} n^2\; 2^{i-1}\left(\beta 2^{2(L-i)} + 2^{2\phi(L-i)}\right)\right) = \\
&= \Theta\left(n^2\left(\sum_{i=1}^{L} \beta 2^{2L-i-1} + \sum_{i=1}^{L} 2^{2\phi L + i(1-2\phi)-1}\right)\right) = \\
&= \Theta\left(n^2\left(\beta 2^{2L}\sum_{i=1}^{L} 2^{-i} + 2^{2\phi L}\sum_{i=1}^{L} 2^{i(1-2\phi)}\right)\right) = \\
&= \Theta\left(n^2\left(\beta k^2\sum_{i=1}^{L} 2^{-i} + k^{2\phi}\sum_{i=1}^{L} 2^{i(1-2\phi)}\right)\right).
\end{aligned} \tag{10}$$

The values of $G_1 = \sum_{i=1}^{L} 2^{-i}$ and $G_2 = \sum_{i=1}^{L} 2^{i(1-2\phi)}$ can be expressed as the sums of L'th first terms of a geometric progressions. In the case of G_1 both initial term and ratio equals 2^{-1}, therefore

$$G_1 = 2^{-1}\,\frac{1-2^{-L}}{1-2^{-1}} = \Theta(1). \tag{11}$$

Similarly, for G_2 both initial term and ratio are $2^{1-2\phi}$ which results in

$$G_2 = 2^{1-2\phi}\,\frac{1-2^{L(1-2\phi)}}{1-2^{1-2\phi}}. \tag{12}$$

When analysing G_2 three cases must be investigated:

(a) When $\phi = \frac{1}{2}$, the sum can be computed explicitly as $G_2 = L = \Theta(\log k)$.

(b) When ϕ is in a neighbourhood of $\frac{1}{2}$, from Eqn. (12) it is apparent that G_2 approaches $\Theta(\log k)$, as $\phi \to \frac{1}{2}$. This can be computed with a use of de L'Hospital rule.

(c) When ϕ is not in a neighbourhood of $\frac{1}{2}$, the denominator from Eqn. (12) can be considered as a constant resulting in $G_2 = \Theta\left(1 + 2^{L(1-2\phi)}\right) = \Theta(1 + k^{1-2\phi})$.

Therefore, in the following analysis we treat G_2 as a function $G_2(k, \phi)$. As a result, the time complexity of the procedure for the perfectly balanced tree is described by the expression

$$T^{\text{final-balanced}}(k, n) = \Theta\left(n^2\left(\beta k^2 + k^{2\phi} G_2(k, \phi)\right)\right). \tag{13}$$

In the perfectly unbalanced tree (case 2) the number of levels corresponding to progressive steps is k. At i'th level (the top is assumed to be level 1) we align a profile of size $k - i$ with a single sequence. The

complexity of the procedure is then

$$\begin{aligned}
&\Theta\left(\sum_{i=1}^{k} T^{\text{profile}}(k-i,1,n)\right) = \\
&= \Theta\left(\sum_{i=1}^{k} n^2 \left(\beta(k-i) + (k-i)^{\phi}\right)\right) = \\
&= \Theta\left(n^2 \sum_{i=1}^{k} \left(\beta i + i^{\phi}\right)\right) = \\
&= \Theta\left(n^2 \left(\beta k^2 + \sum_{i=1}^{k} i^{\phi}\right)\right). \qquad (14)
\end{aligned}$$

The sum

$$G_3 = \sum_{i=1}^{k} i^{\phi} \qquad (15)$$

can be upper bounded as

$$G_3 < \sum_{i=1}^{k} k^{\phi} = k^{\phi+1}. \qquad (16)$$

At the same time, for each $i = k/2, ..., k$ it is true that $i^{\phi} > (k/2)^{\phi}$, thus

$$G_3 > \sum_{i=k/2}^{k} i^{\phi} > \sum_{i=k/2}^{k} (k/2)^{\phi} = (k/2)^{\phi+1}. \qquad (17)$$

From Eqns. (16) and (17) it arises that $G_3 = \Theta(k^{\phi+1})$. As a result the time complexity of the final alignment for the perfectly unbalanced tree is

$$T^{\text{final-unbalanced}}(k,n) = \Theta\left(n^2\left(\beta k^2 + k^{\phi+1}\right)\right). \qquad (18)$$

The following step after alignment construction is refinement. It consists in dividing randomly alignment into two profiles, removing columns consisting only of gaps and realigning them. According to Eqn. (9), the worst case is when profiles are equally long. Taking into account that refinement step is repeated r times, the time complexity is

$$\begin{aligned}
T^{\text{refine}}(k,n) = &\ \Theta\left(r\ T^{\text{profile}}\left(\frac{k}{2},\frac{k}{2},n\right)\right) = \\
= &\ \Theta\left(r\ n^2\left(\beta k^2 + k^{2\phi}\right)\right). \qquad (19)
\end{aligned}$$

Using Eqns. (13), (18) and (19) we can describe the complexity of stage IV in MSAProbs-seq as

$$\begin{aligned}
T^{\text{IV-balanced}}(k,n) = &\ T^{\text{final-balanced}}(k,n) + T^{\text{refine}}(k,n) = \\
= &\ \Theta\left(n^2\left(\beta k^2 + k^{2\phi}G_2(k,\phi) + r\beta k^2 + rk^{2\phi}\right)\right) = \\
= &\ \Theta\left(n^2\left(k^{2\phi}G_2(k,\phi) + r\beta k^2 + rk^{2\phi}\right)\right). \qquad (20)
\end{aligned}$$

for the perfectly balanced tree, and

$$\begin{aligned}
T^{\text{IV-unbalanced}}(k,n) = &\ T^{\text{final-unbalanced}}(k,n) + T^{\text{refine}}(k,n) = \\
= &\ \Theta\left(n^2\left(\beta k^2 + k^{\phi+1} + r\beta k^2 + rk^{2\phi}\right)\right) = \\
= &\ \Theta\left(n^2\left(k^{\phi+1} + r\beta k^2 + rk^{2\phi}\right)\right). \qquad (21)
\end{aligned}$$

for the perfectly unbalanced tree.

Assuming perfect misalignment of input sequences ($\phi = 1$) we use variant (c) of $G_2(k, \phi)$. Therefore, $k^{2\phi} G_2(k, \phi) = \Theta(k + k^2) = \Theta(k^2)$. As a result the time complexity of the procedure is the same for both analysed cases and can be expressed as

$$T^{\text{IV-misalign}}(k, n) = \Theta\left(rk^2 n^2\right). \tag{22}$$

3 MSAProbs

3.1 Posterior matrix calculation

Posterior matrix calculation in MSAProbs algorithm is done according to the inter-task parallelisation scheme. It means, that the smallest portion of computation performed by a thread is a single task. Calculations are preceded by the preparation step in which definitions of all $k(k-1)/2$ posterior tasks are generated serially. Then tasks are distributed among p CPU cores. Taking into account Assumption 1, the time complexity of stage I of MSAProbs is data independent and is

$$\Theta\left(k^2 + \frac{k^2 n^2}{p}\right). \tag{23}$$

Taking into account that $n > p$ (Assumption 4), we obtain

$$T^{\text{I}}(k, n, p) = \Theta\left(\frac{k^2 n^2}{p}\right). \tag{24}$$

The speed-up of MSAProbs stage I over MSAProbs-seq can be computed from Eqns. (1) and (24) as

$$S^{\text{I}}(p) = \Theta(p). \tag{25}$$

As one can see, stage I of MSAProbs scales perfectly with the number of cores independently of the data.

3.2 Tree construction and sequence weighting

Guide tree construction and sequence weighting are performed in the same way as in MSAProbs-seq algorithm. Therefore, the worst and average case time complexities are described by Eqn. (2).

3.3 Consistency transformation

Consistency transformation in MSAProbs algorithm follows the inter-task parallelisation model (each thread processes one or more relaxation tasks). Similarly to the posterior calculation stage, the definitions of all $k(k-1)/2$ tasks are prepared serially and then tasks are distributed among p cores. The scheme of task processing in MSAProbs is identical as in MSAProbs-seq algorithm, and requires $(k-2) \times T^{\text{relax}}(n)$ operations. Taking into account Assumption 1, the time complexity of stage III can be expressed as

$$\Theta\left(ck^2\left(1 + \frac{k\, T^{\text{relax}}(n)}{p}\right)\right). \tag{26}$$

A single relaxation requires accesses to all βn^2 elements of A. As $\beta > \frac{1}{n}$ (Assumption 5) and $n > p$ (Assumption 4) we know that $T^{\text{relax}}(n) > p$ independently of the distribution of sparse rows lengths in A and B. Therefore, the time complexity is

$$T^{\text{III}}(k, n, p) = \Theta\left(\frac{ck^3\; T^{\text{relax}}(n)}{p}\right). \tag{27}$$

From Eqn. (3) this gives the worst case time complexity

$$T^{\text{III-worst}}(k,n,p) = \Theta\left(\frac{c\beta k^3 n^3}{p}\right). \tag{28}$$

Analogously, for the special case of equally long sparse rows described by Eqn. (4) we get

$$T^{\text{III-special}}(k,n,p) = \Theta\left(\frac{c\beta^2 k^3 n^3}{p}\right). \tag{29}$$

From Eqns. (5) and (27) the speed-up of MSAProbs stage III over MSAProbs-seq does not depend on the data and is

$$S^{\text{III}}(p) = \Theta(p). \tag{30}$$

As one can see, stage III of MSAProbs scales perfectly with a number of cores.

3.4 Final alignment construction and refinement

Final alignment construction and refinement are performed in the same way as in MSAProbs-seq algorithm. Therefore, the time complexities for the perfectly balanced and perfectly unbalanced guide tree are described by Eqns.(20) and (21).

4 QuickProbs

4.1 Posterior matrix calculation

Stage I of QuickProbs differs from its MSAProbs counterpart. Firstly, it consists in calculating posterior probability matrices S_{xy} for all $x, y \in U, x \neq y$. Thus, the definition of a posterior task is different and includes three steps, all calculated at GPU. For each sequence pair $x, y \in U, x < y$:

1. calculate dense posterior matrix P_{xy} and pairwise distance d_{xy},
2. build sparse S_{xy} matrix on the basis of P_{xy},
3. build sparse transposed matrix S_{yx} on the basis of P_{xy}.

Secondly, the procedure utilises the intra-task parallelisation scheme. Namely, calculation of a single P_{xy} matrix is done by a single workgroup. Each column of a posterior matrix is computed by a single work-item resulting in $n+1$ work-items in a workgroup. One must however keep in mind that work-items are executed physically at GPU device in wavefronts/warps. Therefore, workgroup size is rounded up to the closest multiplicity of w. As the matrix is calculated anti-diagonally, each work-item executes $2n+1$ iterations. Calculation of P_{xy} requires then $\left\lceil \frac{n}{w} \right\rceil w\ (2n+1)$ operations at GPU. As $n > w$ (Assumption 3) and calculations are distributed among q_2 processing elements, the worst case and average case time complexity of single P_{xy} calculation is

$$\Theta\left(\frac{n^2}{q_2}\right). \tag{31}$$

Because the same processing pattern is used during steps 2 and 3, Eqn. (31) also applies to them. As each posterior task is processed by a single workgroup, there are $k(k-1)/2$ workgroups to be invoked at each step. Taking into account that each workgroup is processed independently by a compute unit, the time complexity of calculating all posterior matrices at GPU is

$$\Theta\left(\left\lceil\frac{k^2}{q_1}\right\rceil \frac{n^2}{q_2}\right) \tag{32}$$

Knowing that $k(k-1)/2 > q_1$ (Assumption 2) we obtain

$$T^{\mathrm{I}}_{\mathrm{GPU}}(k,n,q_1,q_2) = \Theta\left(\frac{k^2\ n^2}{q_1\ q_2}\right). \tag{33}$$

Operations executed at CPU include defining all $k(k-1)/2$ posterior tasks and unpacking calculated S_{xy} matrices to data structures used in QuickProbs. The former is done serially, as in MSAProbs. The latter is distributed among all CPU cores, and its execution time is linear with respect to the number of sparse elements in matrices. As a single matrix contains βn^2 elements, the time complexity of CPU operations in stage I of QuickProbs is

$$\Theta\left(k^2 + \frac{\beta k^2 n^2}{p}\right) \tag{34}$$

which can be simplified using Assumption 4 ($n > p$) and Assumption 5 ($\beta > \frac{1}{n}$) as

$$T^{\mathrm{I}}_{\mathrm{CPU}}(k,n,p) = \Theta\left(\frac{\beta k^2 n^2}{p}\right). \tag{35}$$

As for the memory transfers, QuickProbs passes to GPU all sequences and definitions of all tasks and reads back the calculated sparse matrices. Therefore, the time complexity of transfers is

$$\Theta\left(nk + k^2 + \beta k^2 n^2\right). \tag{36}$$

Taking into account Assumption 5, it reduces to

$$T^{\mathrm{I}}_{\mathrm{MEM}}(k,n) = \Theta(\beta k^2 n^2). \tag{37}$$

Summing up Eqns. (33), (35) and (37), the worst case and average case time complexity of stage I of QuickProbs algorithm is

$$T^{\mathrm{I}}_{\mathrm{GPU}}(k,n,q_1,q_2) + T^{\mathrm{I}}_{\mathrm{CPU}}(k,n,p) + T^{\mathrm{I}}_{\mathrm{MEM}}(k,n) = \Theta\left(\frac{k^2\ n^2}{q_1\ q_2} + \frac{\beta k^2 n^2}{p} + \beta k^2 n^2\right). \tag{38}$$

This simplifies to

$$T^{\mathrm{I}}(k,n,q_1,q_2) = \Theta\left(k^2 n^2 \left(\beta + \frac{1}{q_1 q_2}\right)\right). \tag{39}$$

Using Eqns. (1) and (39) we get the speed-up of QuickProbs stage I over MSAProbs-seq as

$$S^{\mathrm{I}}_{\mathrm{seq}}(q_1,q_2) = \Theta\left(\frac{1}{\beta + \frac{1}{q_1 q_2}}\right) = \Theta\left(q_1 q_2 \frac{1}{\beta q_1 q_2 + 1}\right). \tag{40}$$

If one selects MSAProbs as a reference algorithm, the speed-up calculated on the basis of Eqns. (24) and (39) equals

$$S^{\mathrm{I}}_{\mathrm{par}}(p,q_1,q_2) = \Theta\left(\frac{\frac{1}{p}}{\beta + \frac{1}{q_1 q_2}}\right) = \Theta\left(\frac{q_1 q_1}{p} \frac{1}{\beta q_1 q_2 + 1}\right) \tag{41}$$

Analysis of Eqns. (40) and (41) reveals that for $\beta \to 1$ the speed-ups over MSAProbs-seq and MSAProbs approach, respectively $\Theta(1)$ and $\Theta(1/p)$. This is because the time complexity is dominated by the transfers between CPU and GPU which are performed serially. It is however important to note two facts.

- In our analysis we discarded memory accesses of MSAProbs-seq and MSAProbs as they are an order of magnitude faster than CPU-GPU transfers executed by QuickProbs. However, they are also done serially.

- CPU-GPU transfers, even though slower than CPU memory accesses are much less time consuming than calculations made at the GPU. Thus, even for very large datasets they will not affect execution times.

Taking these into account we think it is reasonable to analyse also the speed-ups of QuickProbs ignoring memory transfers. They are

$$S^{\mathrm{I}}_{\mathrm{seq}}(p, q_1, q_2) = \Theta\left(\frac{1}{\frac{\beta}{p} + \frac{1}{q_1 q_2}}\right) = \Theta\left(q_1 q_2 \frac{1}{\frac{\beta q_1 q_2}{p} + 1}\right). \tag{42}$$

when related to MSAProbs-seq, and

$$S^{\mathrm{I}}_{\mathrm{par}}(p, q_1, q_2) = \Theta\left(\frac{\frac{1}{p}}{\frac{\beta}{p} + \frac{1}{q_1 q_2}}\right) = \Theta\left(\frac{q_1 q_1}{p} \frac{1}{\frac{\beta q_1 q_2}{p} + 1}\right) \tag{43}$$

when MSAProbs is chosen as a reference. When memory transfers are discarded, the time complexity of QuickProbs is dominated by the CPU-parallel procedure of unpacking sparse matrices into data structures used by the algorithm. As a result, for $\beta = 1$ QuickProbs is $\Theta(p)$ times faster than MSAProbs-seq and as fast as MSAProbs. However, in real-life alignment problems $\beta < 1$ (it is usually between 0.05 and 0.1). Moreover, the hidden constant related to the unpacking term is orders of magnitude smaller than for GPU computations. As experiments show, even when aligning in QuickProbs hundreds of sequences of lengths exceeding thousand symbols, unpacking times are negligible when compared to posterior matrices calculation.

4.2 Tree construction and sequence weighting

Guide tree construction and sequence weighting are performed exactly the same as in MSAProbs-seq algorithm. Therefore, the worst and average case time complexities are described by Eqn. (2).

4.3 Consistency transformation

Stage III of QuickProbs consists of two main steps. At first the relaxation of all $S_{xy}, x < y$ matrices over sequences $z \in U, z \neq x, y$ is performed at GPU. Then, the algorithm calculates on CPU transpositions of S'_{xy} generating S'_{yx} matrices. The latter step was not present in the original MSAProbs, thus the definition of a relaxation task in QuickProbs is extended by the calculation of the transposition.

Consistency transformation is performed with a use of intra-task parallelisation model: each task is processed by a single workgroup consisting of many work-items. As previously, the relaxation of S_{xy} matrix over given sequence z consists in performing multiplication of S_{xz} and S_{zy} sparse matrices and adding result to pre-initialised S'_{xy}. The general data processing scheme is the same as in the MSAProbs algorithm, with a difference that S_{xz} rows are processed in horizontal blocks called stripes. There are s_{count} stripes executed concurrently, each having s_{length} sparse elements. An important note is that stripes being processed at the same time are assigned to consecutive rows of S_{xz} matrix, thus at a given moment only one stripe in a row is active. Since each sparse element is analysed by a single work-item, the workgroup size is $s_{\mathrm{length}} \times s_{\mathrm{count}}$. Additionally, row lengths and number of rows are rounded up to the closest multiplicities of s_{length} and s_{count}, respectively. Several things have to be taken into consideration when setting s_{length} and s_{count}. First of all, $s_{\mathrm{length}} \times s_{\mathrm{count}}$ should be a multiplicity of wavefront/warp size w and be sufficiently large to provide good GPU occupancy. On the other hand, the larger s_{length}, the more work items idle when last stripe of a row is processed. Similarly, the larger s_{count}, the more rows are being analysed concurrently and the more divergence in kernel executions caused by different stripe row lengths can be observed. This results in reduced performance. Therefore, we assume that $s_{\mathrm{length}} \leq w$ and $s_{\mathrm{count}} \leq w$, while $s_{\mathrm{length}} \times s_{\mathrm{count}} = iw$, for some positive integer i.

Another difference from MSAProbs algorithm is that due to limited memory resources on GPU devices, S'_{xy} matrices are processed by QuickProbs directly in a sparse form. Therefore, the algorithm needs to find a sparse element to be updated before the addition is made. This is done with a use of binary search. For simplicity let $S'_{xy} = C$, $S_{xz} = A$, $S_{zy} = B$ and $A(i, \Box)$ denote i'th row of A.

As previously, the number of accesses to B is maximal when A contains n non-empty rows of length βn while B contains βn non-empty rows of length n. Moreover, A rows have to be identical and their elements have to point to all non-empty B rows. However, in contrast to MSAProbs-seq and MSAProbs, for each B access we need to find with a use of binary search an element in a row of C with the same column index. Therefore, in the worst case C has the same structure as A and contains n non-empty rows of length βn. The number of operations needed to perform matrix multiplication in the above-mentioned case is

$$\Theta\left(\lceil n\rceil_{s_{\text{count}}} \lceil \beta n\rceil_{s_{\text{length}}} \lceil n\rceil_{s_{\text{length}}} \log n\right) \tag{44}$$

Taking into account assumptions concerning number of stripes and their length and knowing that the procedure is distributed among q_2 processing elements in a compute unit, we obtain the worst case time complexity

$$T^{\text{relax-worst}}(n, q_2) = \Theta\left(\frac{n^2 \lceil \beta n\rceil_{s_{\text{length}}} \log n}{q_2}\right). \tag{45}$$

Actually, for small problems it may happen that $q_2 > \beta n^2$ and the denominator of Eqn. (45) should be $\min(q_2, \beta n^2)$. However, as one compute unit can concurrently run several workgroups if their sizes are smaller than q_2, the simplification we made will not affect overall time complexity of the consistency transformation. As in MSAProbs-seq we also analyse the special case, where all rows in A, B and C matrices are of equal length βn. In such situation, number of operations is

$$\Theta\left(\lceil n\rceil_{s_{\text{count}}} \lceil \beta n\rceil^2_{s_{\text{length}}} \log n\right) \tag{46}$$

resulting in the following time complexity

$$T^{\text{relax-special}}(n, q_2) = \Theta\left(\frac{n \lceil \beta n\rceil^2_{s_{\text{length}}} \log n}{q_2}\right). \tag{47}$$

There are $k(k-1)/2$ tasks to be done in the consistency transformation, each performing $k-2$ multiplications. Matrices are processed independently on q_1 compute units. Taking into account that $k(k-1)/2 > q_1$ (Assumption 2), the time complexity of the relaxation of all matrices over all sequences is

$$T^{\text{III}}_{\text{GPU}}(k, n, q_1, q_2) = \Theta\left(\frac{k^3\ T^{\text{relax}}(n, q_2)}{q_1}\right). \tag{48}$$

Procedures executed at the CPU include preparing descriptions of all relaxation tasks, unpacking output S'_{xy} matrices to structures used in QuickProbs and calculating transposed sparse matrices S'_{yx}. The former action is executed serially. The two latter are distributed among p cores using inter-task parallelisation scheme. As time required for a single unpacking and transposition is proportional to the number of sparse elements, the worst and the average case time complexity of steps executed at CPU is

$$\Theta\left(k^2 + \frac{\beta k^2 n^2}{p}\right). \tag{49}$$

As $n > p$ (Assumption 4) and $\beta > \frac{1}{n}$ (Assumption 5) we obtain

$$T^{\text{III}}_{\text{CPU}}(k, n, p) = \Theta\left(\frac{\beta k^2 n^2}{p}\right). \tag{50}$$

Regarding memory transfers, QuickProbs passes to GPU descriptions of all tasks and sparse matrices. It reads back sparse matrices after relaxation. Therefore, the worst and also the average case time complexity of transfers is

$$\Theta\left(k^2 + \beta k^2 n^2\right). \tag{51}$$

Using once again Assumption 5 this reduces to

$$T^{\text{III}}_{\text{MEM}}(k,n) = \Theta\left(\beta k^2 n^2\right). \tag{52}$$

The time complexity of a single consistency transformation is obtained by summing Eqns. (48), (50) and (52)

$$T^{\text{III}}_{\text{GPU}}(k,n,q_1,q_2) + T^{\text{III}}_{\text{CPU}}(k,n,p) + T^{\text{III}}_{\text{MEM}}(k,n) = \Theta\left(\frac{k^3\ T^{\text{relax}}(n,q_2)}{q_1} + \frac{\beta k^2 n^2}{p} + \beta k^2 n^2\right). \tag{53}$$

Taking into account that the consistency transformation is executed c times we get

$$T^{\text{III}}(k,n,q_1,q_2) = \Theta\left(ck^2\left(\beta n^2 + \frac{k\ T^{\text{relax}}(n,q_2)}{q_1}\right)\right). \tag{54}$$

The speed-up of stage III of QuickProbs with respect to MSAProbs-seq can be calculated on the basis of Eqns. (5) and (54) as

$$S^{\text{III}}_{\text{seq}}(k,n,q_1,q_2) = \Theta\left(\frac{k\ T^{\text{relax}}(n)}{\beta n^2 + \frac{k\ T^{\text{relax}}(n,q_2)}{q_1}}\right). \tag{55}$$

From Eqns. (3) and (45) in the worst case this is

$$S^{\text{III-worst}}_{\text{seq}}(k,n,q_1,q_2) = \Theta\left(\frac{\beta k n^3}{\beta n^2 + \frac{kn^2\lceil\beta n\rceil_{s_{\text{length}}}\log n}{q_1 q_2}}\right) = \Theta\left(q_1 q_2 \frac{1}{\frac{q_1 q_2}{kn} + \frac{\lceil\beta n\rceil_{s_{\text{length}}}}{\beta n}\log n}\right). \tag{56}$$

Analogously, on the basis of Eqns. (4) and (47) we calculate speed-up for the special case of equally long sparse rows as

$$S^{\text{III-special}}_{\text{seq}}(k,n,q_1,q_2) = \Theta\left(\frac{\beta^2 k n^3}{\beta n^2 + \frac{kn\lceil\beta n\rceil^2_{s_{\text{length}}}\log n}{q_1 q_2}}\right) = \Theta\left(q_1 q_2 \frac{1}{\frac{q_1 q_2}{\beta kn} + \left(\frac{\lceil\beta n\rceil_{s_{\text{length}}}}{\beta n}\right)^2\log n}\right). \tag{57}$$

As one can see in both cases the speed-ups are proportional to $q_1 q_2$ worsened by some factor. This factor consists of two components. The first one corresponds to the cost of transfers between GPU and CPU memory and equals $\frac{q_1 q_2}{kn}$ and $\frac{q_1 q_2}{\beta kn}$ for the worst and the special case, respectively. It is apparent then that the importance of the transfers decreases with increasing kn and is greater for the special case when GPU computations are faster (note $\beta \leq 1$). The second component represents:

- effect of rounding sparse rows length to the multiplicity of stripe length: $\frac{\lceil \beta n \rceil_{s_\text{length}}}{\beta n}$,
- the complexity of binary search in C matrix: $\log n$.

The rounding effect is more meaningful in the special case of equally long sparse rows as the coefficient is squared.

If one chooses MSAProbs as a reference algorithm, the speed-up calculated with a use of Eqns. (27) and (54) is

$$S_\text{par}^\text{III}(k, n, p, q_1, q_2) = \Theta\left(\frac{\frac{k\ T^\text{relax}(n)}{p}}{\beta n^2 + \frac{k\ T^\text{relax}(n, q_2)}{q_1}}\right). \tag{58}$$

From Eqns. (3) and (45) in the worst case this gives

$$\begin{aligned} S_\text{par}^\text{III-worst}(k, n, p, q_1, q_2) &= \Theta\left(\frac{\frac{\beta k n^3}{p}}{\beta n^2 + \frac{k n^2 \lceil \beta n \rceil_{s_\text{length}} \log n}{q_1 q_2}}\right) = \\ &= \Theta\left(\frac{q_1 q_2}{p} \frac{1}{\frac{q_1 q_2}{kn} + \frac{\lceil \beta k n \rceil_{s_\text{length}}}{\beta n} \log n}\right). \end{aligned} \tag{59}$$

Similar analysis can be performed in the special case. From Eqns. (4) and (47) we get

$$\begin{aligned} S_\text{par}^\text{III-special}(k, n, p, q_1, q_2) &= \Theta\left(\frac{\frac{\beta^2 k n^3}{p}}{\beta n^2 + \frac{k n \lceil \beta n \rceil^2_{s_\text{length}} \log n}{q_1 q_2}}\right) = \\ &= \Theta\left(\frac{q_1 q_2}{p} \frac{1}{\frac{q_1 q_2}{\beta k n} + \left(\frac{\lceil \beta n \rceil_{s_\text{length}}}{\beta n}\right)^2 \log n}\right). \end{aligned} \tag{60}$$

Speed-ups over MSAProbs are proportional to $\frac{q_1 q_2}{p}$ and are worsened by the same factors as speed-ups related to MSAProbs-seq.

4.4 Final alignment construction and refinement

Let X and Y be the profiles to be processed; k_1, k_2 indicate their sizes and n_1, n_2 their lengths. As previously, the first step is to compute posterior matrix P_{XY} by accumulating elements of all pairwise posterior matrices S_{xy} such that $x \in X$ and $y \in Y$. In QuickProbs, however, this procedure is distributed among p cores, each being responsible for accumulation of $\frac{k_1 k_2}{p}$ sparse matrices. As a consequence, each thread needs to store its own copy of P_{XY}. Then copies are merged in parallel and the dynamic programming procedure on output matrix is executed serially. Both of these procedures require $\Theta(n_1 n_2)$ operations. The resulting time complexity is then

$$T^\text{profile}(k_1, k_2, n_1, n_2, n) = \Theta\left(\frac{\beta k_1 k_2 n^2}{p} + n_1 n_2\right). \tag{61}$$

If we express profile lengths with a help of ϕ function, the complexity of aligning two profiles becomes

$$T^\text{profile}(k_1, k_2, n) = \Theta\left(n^2\left(\frac{\beta k_1 k_2}{p} + k_1^\phi k_2^\phi\right)\right). \tag{62}$$

As in the case of MSAProbs-seq algorithm, the time complexity of full alignment construction depends on the guide tree structure. Analogously to Eqn. (13), in the case of perfectly balanced tree it is

$$T^{\text{final-balanced}}(k,n,p) = \Theta\left(n^2\left(\frac{\beta k^2}{p} + k^{2\phi}G_2(k,\phi)\right)\right). \tag{63}$$

When the perfectly unbalanced case is of interest as in Eqn. (18), we get

$$T^{\text{final-unbalanced}}(k,n,p) = \Theta\left(n^2\left(\frac{\beta k^2}{p} + k^{\phi+1}\right)\right). \tag{64}$$

The same parallelisation pattern is used in the case of iterative refinement resulting in

$$T^{\text{refine}}(k,n,p) = \Theta\left(rn^2\left(\frac{\beta k^2}{p} + k^{2\phi}\right)\right). \tag{65}$$

The time complexity of stage IV of QuickProbs for the perfectly balanced guide tree is

$$T^{\text{IV-balanced}}(k,n,p) = \Theta\left(n^2\left(k^{2\phi}G_2(k,\phi) + \frac{r\beta k^2}{p} + rk^{2\phi}\right)\right) \tag{66}$$

and for the perfectly unbalanced we get

$$T^{\text{IV-unbalanced}}(k,n,p) = \Theta\left(n^2\left(k^{\phi+1} + \frac{r\beta k^2}{p} + rk^{2\phi}\right)\right). \tag{67}$$

As in MSAProbs-seq, in the case of misalignment ($\phi = 1$) the time complexity of the stage is the same for balanced and unbalanced case and can be expressed as

$$T^{\text{IV-misalign}}(k,n) = \Theta\left(rk^2n^2\right). \tag{68}$$

The speed-up QuickProbs over MSAProbs-seq and MSAProbs for the balanced tree can be calculated from Eqns. (20) and (66) as

$$S^{\text{IV-balanced}}(k,p) = \Theta\left(\frac{k^{2\phi}G_2(k,\phi) + r\beta k^2 + rk^{2\phi}}{k^{2\phi}G_2(k,\phi) + \frac{r\beta k^2}{p} + rk^{2\phi}}\right) \tag{69}$$

Analogously, from (21) and (67) we can derive the speed-up for the unbalanced tree as

$$S^{\text{IV-unbalanced}}(k,p) = \Theta\left(\frac{k^{\phi+1} + r\beta k^2 + rk^{2\phi}}{k^{\phi+1} + \frac{r\beta k^2}{p} + rk^{2\phi}}\right). \tag{70}$$

In the misalignment situation ($\phi = 1$) the speed-up in both cases reduces to

$$S^{\text{IV-misalign}} = \Theta(1). \tag{71}$$

This is because the stage is dominated by performing dynamic programming over profile-profile posterior matrices which in all algorithms is done serially. However, $\phi = 1$ implies that all sequences in the result are misaligned, i.e., the length of output alignment is kn, which is very unrealistic situation. In practical problems ϕ is much lower than 1 and execution times of both serial and parallel variants are dominated by the middle components of Eqns. (20), (21), (66), and (67). As a result, obtained speed-up is $\Theta(p)$ which was suggested by our experiments.

5 Discussion

In the supplement we performed a detailed time complexity analysis of three algorithms: MSAProbs-seq, MSAProbs and QuickProbs. The former is a serial variant of MSAProbs in which we removed all operations needless for sequential execution. In MSAProbs there are two stages run in parallel on CPU: posterior matrices calculation (stage I) and consistency transformation (stage III). Both of them utilise inter-task concurrency model (a thread is responsible for processing of one or more tasks). As a result speed-ups over MSAProbs are ideal and equal $\Theta(p)$ with respect to the number of cores p and are independent of the data.

The different situation is for QuickProbs in which both, posterior matrices calculation and consistency transformation were customised for GPU execution with a use of intra-task model (a task is processed by several work-items). Additionally, the alignment construction and refinement (stage IV) were parallelised on CPU. Analysing stage I, it becomes apparent that for very dense posterior matrices ($\beta \to 1$) the time complexity of the stage is dominated by the transfers between CPU and GPU memory which are performed serially. This results in the speed-ups over MSAProbs-seq and MSAProbs approaching respectively, $\Theta(1)$ and $\Theta(1/p)$ independently of the data. However, for several reasons overhead introduced by transfers and operations performed at CPU is negligible with respect to GPU computations. Experiments on very large real-life datasets confirm that speed-ups are in practice much better. As for the consistency transformation stage, the QuickProbs time complexity is dominated by GPU computations. We analysed here the worst case and the special case of equally long sparse rows. The general observations hold for both of them. The speed-ups over MSAProbs-seq and MSAProbs are proportional to $q_1 q_2$ and $\frac{q_1 q_2}{p}$, respectively, worsened by some factor. The factor represents the effect of rounding sparse row lengths to the multiplicity of stripe length and the execution time of binary search performed on rows of relaxed matrix. Regarding the last QuickProbs stage, the worst case speed-up is $\Theta(1)$. However, this case assumes very unrealistic situation when all sequences are misaligned, i.e., the output alignment is of length kn. In practice, the execution time is dominated by the term which was a subject of parallelisation resulting in $\Theta(p)$-fold speed-up.
